# JointTracker: Real-time inertial kinematic chain tracking with joint position estimation

**DOI:** 10.12688/openreseurope.16939.2

**Published:** 2025-01-22

**Authors:** Bertram Taetz, Michael Lorenz, Markus Miezal, Didier Stricker, Gabriele Bleser-Taetz

**Affiliations:** 1 Augmented Vision, German Research Center for Artificial Intelligence, Kaiserslautern, Rhineland-Palatinate, 67663, Germany; 2 IT & Engineering, International University of Applied Sciences, Erfurt, Thuringia, 99084, Germany

**Keywords:** inertial motion capture, 3D kinematics, 3D human pose estimation, online joint position estimation, calibration-free, recursive state estimation, parameter estimation

## Abstract

In-field motion capture is drawing increasing attention due to the multitude of application areas, in particular for human motion capture (HMC). Plenty of research is currently invested in camera-based markerless HMC, however, with the inherent drawbacks of limited field of view and occlusions. In contrast, inertial motion capture does not suffer from occlusions, thus being a promising approach for capturing motion outside the laboratory. However, one major challenge of such methods is the necessity of spatial registration. Typically, during a predefined calibration sequence, the orientation and location of each inertial sensor are registered with respect to an underlying skeleton model. This work contributes to calibration-free inertial motion capture, as it proposes a recursive estimator for the simultaneous online estimation of all sensor poses and joint positions of a kinematic chain model like the human skeleton. The full derivation from an optimization objective is provided. The approach can directly be applied to a synchronized data stream from a body-mounted inertial sensor network. Successful evaluations are demonstrated on noisy simulated data from a three-link chain, real lower-body walking data from 25 young, healthy persons, and walking data captured from a humanoid robot.

## 1 Introduction

Motion capture, in particular human motion capture (HMC), is a well-studied field with multiple application areas like healthcare, sports ^1, 2^, human-machine interaction ^3^, workplace analysis ^4, 5^, robotics, teleworking, additive manufacturing and human safety ^6^. Numerous HMC methods utilize camera-based approaches, which, however, face limitations such as occlusions caused by external factors or self-occlusions. Inertial HMC (IHMC) does not suffer from occlusions and is a promising approach for in-field measurements. It uses multiple inertial sensors (inertial measurement units, IMUs) mounted on body segments, using e.g., straps or clothing integration ^7– 9^. The motion is typically deduced via a suitable state estimation approach in combination with a personalized biomechanical model ^10^. IHMC is an approach for in-field HMC that does not suffer from occlusions. However, it comes with other challenges, such as integration drift ^11^, sensitivity to magnetic disturbances ^12^ or calibration issues. The latter are typically related to segment lengths and sensor poses with respect to the anatomical reference frames of the associated body segments, the so-called IMU-to-segment (I2S) calibrations ^13– 16^. Some of the mentioned challenges are connected, e.g. omitting magnetometer measurements can lead to orientation drift, while magnetometer usage can violate the often-used constant magnetic field assumption and lead to incorrect state estimates. The I2S calibration is a major concern for assessing and monitoring anatomical joint angles since I2S orientation errors linearly propagate into joint-angle errors ^13^. I2S calibration is currently considered the main challenge for IHMC in biomechanical motion analysis ^17^, and an automated (self-calibrating) approach can be regarded as crucial for reliable in-field usage of the inertial tracking technology. Self-calibrating IHMC approaches include the direct I2S alignment estimation. Some promising approaches for I2S orientation and position calibration for different body parts are proposed in 14, 15, 18, 19. Direct estimation of biomechanical joint angles without I2S alignment is proposed in 20, 21, but via exploiting a specific calibration motion and assumptions / heuristics concerning the human skeleton and walking kinematics.

**Figure 1.**
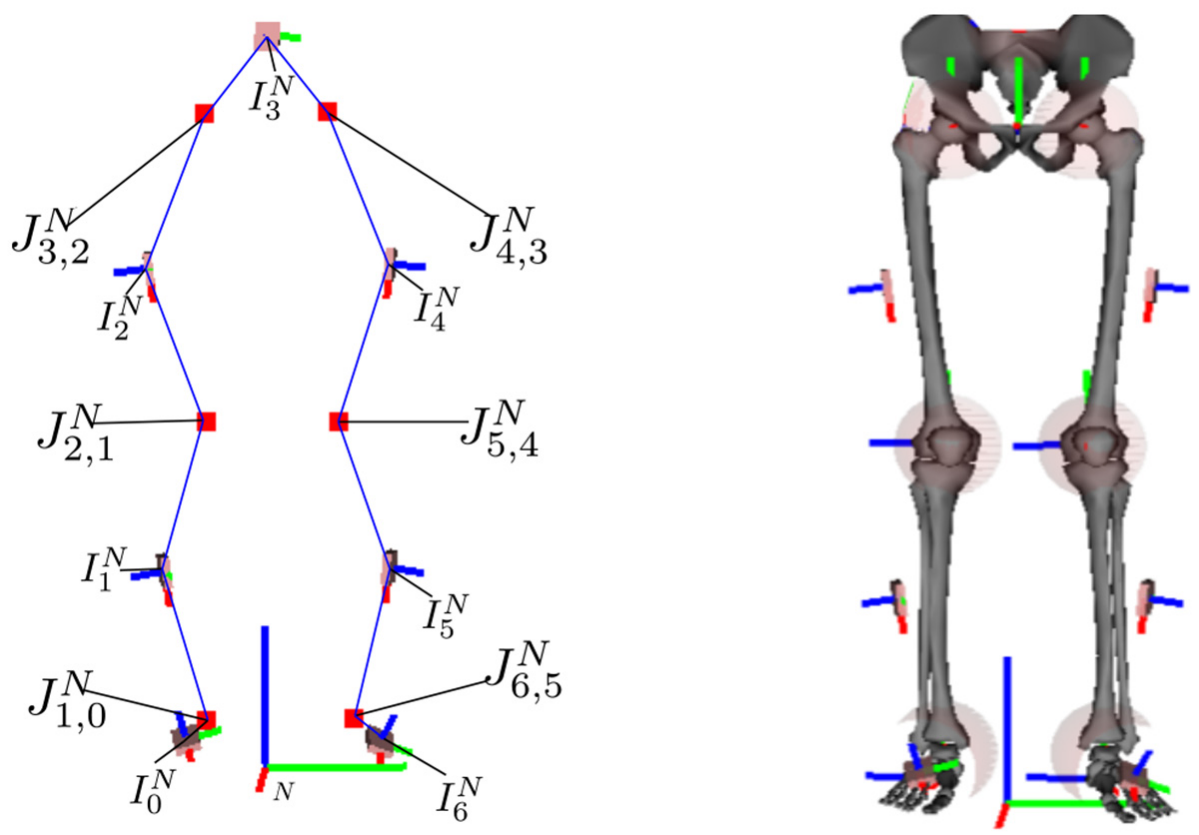
The left image is an illustration of the kinematic model with joints (red squares), IMU poses, and connections between IMU and joint centers (blue lines), which is tracked using the proposed approach. The right image shows the corresponding biomechanical skeleton model with I2S calibrations, based on 24, where the joints are emphasized with semi-transparent spheres.

This work derives and proposes an algorithm for self-calibrating inertial motion capture based on a general kinematic chain model representation consisting of global IMU poses with local (IMU-centered) joint positions, as shown in Figure 1 (left). The model is applicable to any kinematic chain and not restricted to or specialized to a human skeleton. Note, joint position refers here to the actual positional offset between joint and adjacent IMU, not to joint angles. Hence, the focus is on self-calibration of the joint positions in the IMU frames or, in other words, on a calibration-free approach with respect to the I2S orientations and positions. The proposed algorithm can be used to track joint positions, i.e. joint trajectories, for motion analysis, and it delivers consistent information with camera-based markerless 3D joint tracking, where the majority of approaches relies on joint position detections on the image plane (2D) with a subsequent lifting of the joint positions into 3D space ^22^. The body pose can then be illustrated by connecting the segments at the joints, which is common practice in the vision community ^23^, and which can be done with the tracking results of our proposed algorithm as well. Moreover, the proposed algorithm allows estimating IMU trajectories in one coordinate system with minimal magnetometer usage (usually one measurement sample only for initialization). For the skeleton state estimation and joint parameter identification, we propose a consistent recursive estimation of IMU poses in a global coordinate frame and joint positions in the IMU coordinate frames.

Previous approaches to joint position estimation typically work offline, independently per joint and exploit the measured accelerations ^18, 25, 26^. A recent integration-based (using velocities and positions) example operating on a complete kinematic chain is 20. It estimates IMU poses, multiple joint positions, and hinge axes, however, supported by segment length information and in an offline manner. In contrast to these methods, our integration-based approach works online for a complete kinematic chain without requiring prior information. Moreover, we also investigate an indicator for convergence of the joint position parameters, which none of the previous approaches did.

In summary, the main contributions of this work are:

- mathematical derivation of a consistent optimization-based recursive (real-time capable) state estimator, which uses only data from the current timestep, for
- calibration-free simultaneous online estimation of IMU kinematics and joint (or fixed) positions for three degrees of freedom (DoF) joints for kinematic chain models (so that the chain pose can be illustrated by connecting the segments at the joints). This results in online segment length estimation for all segments connecting two joints.
- convergence indicator for the motion-dependent joint position parameters
- minimal magnetometer usage (only for initialization).

In the following, Section 2 introduces the mathematical notation and derives the recursive state estimator, including the corresponding models, while also addressing initialization and observability. Section 3 evaluates the method on three different experimental setups: 1) simulated IMU data from a three-link chain fixed in space, 2) real lower-body walking data from a group of young, healthy persons, and 3) real lower-body walking data from a humanoid robot. Section 4 concludes this work and discusses open problems and further research.

## 2 Methods

**Figure 2.**
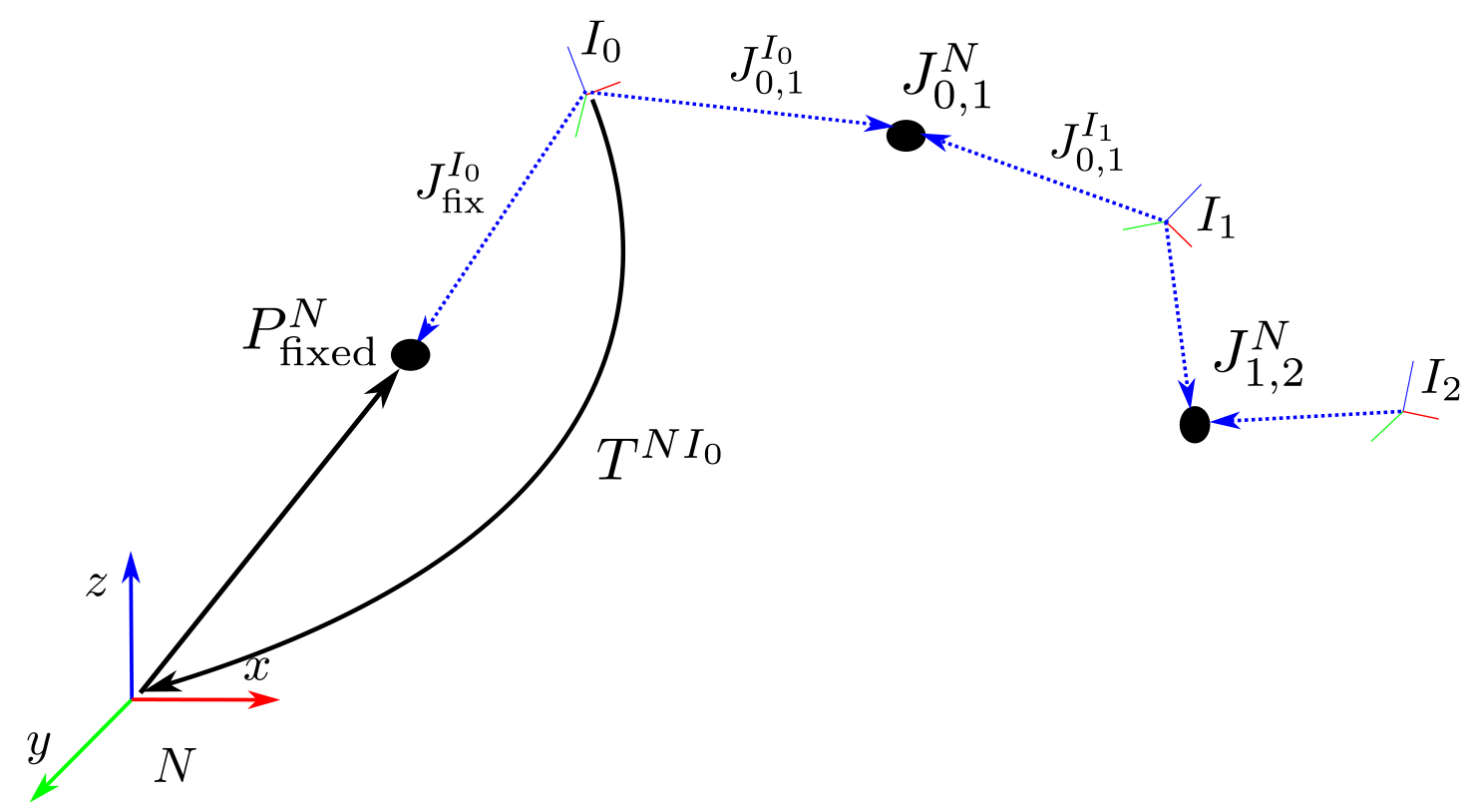
Coordinate systems and notation.

In this section, we present the notation ( Section 2.1), the problem statement, where a batch estimator is defined ( Section 2.2), the derivation of the optimization-based recursive state estimator from the batch estimator ( Section 2.3), and the motion ( Section 2.4) and measurement models ( Section 2.5) used to estimate the state of the kinematic chain model, illustrated in Figure 1 and Figure 2. Note, the mathematical derivation of the recursive state estimator extends the work presented in 27 for a nonlinear motion model and is a theoretical contribution of this article.

### 2.1 Notation

The following description uses Figure 2 as a reference illustration. The navigation frame *N* has its *z*-axis aligned with gravity, and its *x*-axis pointing towards magnetic north. It is the reference frame to which all IMUs are registered. Each IMU *i* ∈ *ℐ*, | *ℐ*| = *N _I_*, has its own coordinate system, *I _i_*, which is related to the navigation frame via a pose transformation, e.g., using a homogeneous matrix


$$\;T^{NI_{i}}=\left(\begin{array}{c} R^{NI_{i}}\\ 0_{3}^{T} \end{array}\;\begin{array}{c} I_{i}^{N}\\ 1 \end{array}\right).\;(1)$$

Here, $I_{i}^{N}$ ∈ ℝ ^3^ denotes the position of the IMU in the navigation frame. The orientation matrix *R ^NI _i_^* ∈ (3) denotes the IMU rotation relative to the navigation frame. Hence, a vector that is right multiplied to *R ^NI _i_^* is transformed from *I _i_* to *N*. The inverse of the homogeneous matrix is


$$\;{\left(T^{NI_{i}}\right)}^{-1}=T^{I_{i}N}=\left(\begin{array}{cc} R^{I_{i}N} & -R^{I_{i}N}I^{N}\\ 0_{3}^{T} & 1 \end{array}\right).\;(2)$$

The inverse rotation matrix is the transpose and denoted by ( *R ^NI _i_^*) ^*T*^ = *R ^I _i_N^*. As rotation parametrizations we use Modified Rodriguez Parameters (MRP) *χ*, unit quaternions *q* = ( *q*_0_, *q*_1_, *q*_2_, *q*_3_), where *q*_0_ represents the scalar part, and rotation matrices *R*. Conversions are indicated with e.g. *χ*( *q*), refer to 28 for the equations. The position of a joint connecting two neighbouring IMUs *I _i_* and *I _j_* is denoted in the navigation frame by $J_{i,j}^{N}$ , ( *i*, *j*) ∈ , the set of all joint indices. This joint position is, however, estimated separately in the frames of the two adjacent IMUs as $J_{i,j}^{I_{i}}$ and $J_{i,j}^{I_{j}}$ . For our first test case of a simulated three link chain, one segment is connected to the environment with a fixed point, which is also estimated. The indices for additional (optional) fixed point estimates, $J_{\text{fix}}^{I_{i}}$ are collected in *i* ∈ *ℱ*.

The symbol *X _t_* denotes the collection of all hidden states to be estimated at time step *t* and *X*_1: *N _T_*_ = ${\left\{X_{t}\right\}}_{t=1}^{N_{T}}$ denotes a complete time sequence of states consisting of *N _T_* time instances. Hence, our kinematic model is defined via IMUs and connecting joints only.

This should not be confused with a biomechanical model, where segments *S _i_* are defined via anatomical landmarks and are associated with the respective IMUs, typically via a rigid I2S transformation *T ^S _i_ I _i_^*. The latter would need an I2S rotation calibration ( *R ^S _i_ I _i_^*) in addition to the estimates given via the proposed algorithm. Note that the I2S position could be computed from the joint position estimates in case the I2S rotation would be known, via *I ^S _i_^* = − *R ^S _i_I _i_^* $J_{i,j}^{I_{i}}$ . The associated biomechanical skeleton could then be directly computed via *T ^NS _i_^* = *T ^NI _i_^ T ^I _i_S _i_^*. A lower body skeleton with respective joints and IMUs is illustrated in Figure 1 (left). Figure 1 (right) shows a corresponding biomechanical skeleton with a given I2S alignment (which is, however, not needed or estimated by the proposed algorithm).

### 2.2 Problem statement

Synchronized measurements from a network with *N _I_* IMUs at time *t* are denoted


$$\;Y_{t}=\left({\left\{\begin{array}{c} y_{a,t}^{I_{i}}\\ y_{\omega ,t}^{I_{i}}\\ y_{m,t}^{I_{i}} \end{array}\right\}}_{i\in ℐ}\right)\;.\;(3)$$

Here, $y_{a,t}^{I_{i}}$ ∈ ℝ ^3^ is the accelerometer measurement, $y_{\omega ,t}^{I_{i}}$ ∈ ℝ ^3^ is the gyroscope measurement, and $y_{m,t}^{I_{i}}$ ∈ ℝ ^3^ is the magnetometer measurement, all in the IMU frame *I _i_*. We consider a sensor network with | *ℐ*| > 1 IMUs connecting | | ≥ 1 joints. Moreover, we assume one IMU being mounted on each segment of the considered kinematic chain.

The aim is to recursively estimate the time dependent state of a kinematic chain of *N _I_* IMUs together with the joint and optionally fixed position parameters. For each IMU *i*, the state includes its position $I_{i,t}^{N}$ velocity ${\dot{I}}_{i,t}^{N}$ , linear acceleration ${\ddot{I}}_{i,t}^{N}$ , orientation $\chi_{i,t}^{IN}$ , and angular velocity $\omega_{I_{i},t}^{NI_{i}}$ , all with respect to the navigation frame *N* and element of ℝ ^3^. Together with the previously mentioned IMU centered joint and fixed position parameters, the state can be written as


$$\;X_{t}={\left(\begin{array}{c} {\left\{\begin{array}{c} \omega_{I_{i}}^{NI_{i}}\\ {\ddot{I}}_{i}^{N} \end{array}\right\}}_{i\in ℐ}\\ {\left\{\begin{array}{c} \chi^{NI_{i}}\\ I_{i}^{N}\\ {\dot{I}}_{i}^{N} \end{array}\right\}}_{i\in ℐ}\\ {\left\{\begin{array}{c} J_{\left(i,j\right)}^{I_{i}}\\ J_{\left(i,j\right)}^{I_{j}} \end{array}\right\}}_{(i,j)\in J}\\ {\left\{J_{\text{fix}}^{I_{i}}\right\}}_{i\in ℱ} \end{array}\right)}_{t}.\;(4)$$

where the specific grouping will be explained later.

Mathematically speaking, we want to obtain a maximum a-posteriori (MAP) state estimate, given the sensor measurements from ( 3) and statistical models (priors) regarding the IMU motion and the kinematic structure of the skeleton (joint connections). To obtain a formal estimation problem, we utilize Bayes’ theorem and the fact that a monotonic function does not change the estimates for extreme points. Thus, the estimation objective can be written as


$$\;\begin{array}{c} \begin{array}{l} {\hat{X}}_{1:N_{T}}=\underset{X_{1:N_{T}}}{\text{argmax}\;}p(X_{1:N_{T}}\text{|}Y_{1:N_{T}})\\ \;=\underset{X_{1:N_{T}}}{\text{argmin}}-\text{log}(p(Y_{1:N_{T}}\text{|}X_{1:N_{T}}))-\text{log}(p(X_{1:N_{T}}))+\text{log}(p(Y_{1:N_{T}})). \end{array} \end{array}\;(5)$$

Note, additional parameter estimates, like sensor biases, often denoted as *θ* ^29^, could be included, but are left out for brevity of notation. Using the Markov property, the prior can be further subdivided into a prior distribution at the first time step and a temporal prior between subsequent time steps, i.e.


$$\;\text{log}(p(X_{1:N_{T}}))=\text{log}(p(X_{1}))+\sum_{t=2}^{N_{T}}\log(p(X_{t}\text{|}X_{t-1})).\;(6)$$

Assuming independent measurements and applying the Markov assumption also to the likelihood, the objective becomes


$$\;{\hat{X}}_{1:N_{T}}=\underset{X_{1:N_{T}}}{\text{argmin}}\underset{\text{likelihood}}{\underset{︸}{-\sum_{t=1}^{N_{T}}\log(p(Y_{t}\text{|}X_{t}))}}\underset{\text{initial}\;\text{prior}}{\underset{︸}{-\log(p(X_{1}))}}\underset{\text{motion}\;\text{prior}}{\underset{︸}{-\sum_{t=2}^{N_{T}}\log(p(X_{t}\text{|}X_{t-1}))}}+\underset{\text{constant}}{\underset{︸}{\text{log}(p(Y_{1:N_{T}}\text{))}}}\text{.}\;(7)$$

The following state splitting is used in this work:


$$\;X_{t}=\left\{\begin{array}{c} X_{t}^{1}\\ X_{t}^{2} \end{array}\right\},\;\;\text{with}\;X_{t}^{1}={\left({\left\{\begin{array}{c} \omega_{I_{i}}^{NI_{i}}\\ {\ddot{I}}_{i}^{N} \end{array}\right\}}_{i\in ℐ}\right)}_{t},\;X_{t}^{2}={\left(\begin{array}{c} {\left\{\begin{array}{c} \chi^{NI_{i}}\\ I_{i}^{N}\\ {\dot{I}}_{i}^{N} \end{array}\right\}}_{i\in ℐ}\\ {\left\{\begin{array}{c} J_{i,j}^{I_{i}}\\ J_{i,j}^{I_{j}} \end{array}\right\}}_{(i,j)\in J}\\ {\left\{J_{\text{fix}}^{I_{i}}\right\}}_{i\in ℱ} \end{array}\right)}_{t}.\;(8)$$

The splitting is motivated by the following discrete state-space model


$$\;X_{t}^{1}=F_{t}^{1}(X_{t-1})+w_{t},\;(9)$$

$$\;X_{t}^{2}=F_{t}^{2}(X_{t-1}),\;(10)$$

$$\;Y_{t}=H_{t}(X_{t})+v_{t},\;(11)$$

where *w _t_* ~ *N* (0, *Q _t_*), *v _t_* ~ *N*(0, Σ *_t_*) denote normally distributed additive process and measurement noises for the motion model $F_{t}^{1}$ and the measurement model *H _t_*. Note that there is no process noise modeled for the states in $X_{t}^{2}$ . The time-dependent states $\omega_{I_{i}}^{NI_{i}}$ and ${\ddot{I}}_{i}^{N}$ have directly associated measurement models (see Section 2.5), and thus a process noise is crucial in the model. The joint and fixed positions, $J_{\left(i,j\right)}^{I_{i}},J_{\left(i,j\right)}^{I_{j}},J_{\text{fix}}^{I_{i}}$ , are modeled as parameters. Since they are linear with respect to the other states in the joint ( 49) and velocity ( 50) connection models, their estimation can be viewed as a linear Bayesian regression jointly estimated with the other states. The remaining states in $X_{t}^{2}$ are time-dependent but do not have an associated measurement model. Thus, they need to satisfy the proposed motion model strictly to avoid increasing estimation noise. The proposed subdivision is crucial to obtain a consistent and long-term stable recursive estimator (see Section 2.3). Leaving out the constants, the estimation problem with the model Equation (9) to Equation (11) can generally be written as the following constrained non-linear weighted least squares problem


$$\;\begin{array}{l} {\hat{X}}_{1:N_{T}}=\underset{X_{1:N_{T}}}{\min}\frac{1}{2}\text{||}X_{1}-{\hat{X}}_{1}\text{|}{\text{|}}_{P_{1}^{-1}}^{2}+\frac{1}{2}\sum_{t=2}^{N_{T}}\sum_{i\in ℐ}\text{||}w_{t}\text{|}{\text{|}}_{Q_{t}^{-1}}^{2}+\frac{1}{2}\sum_{t=1}^{N_{T}}\sum_{i\in ℐ}\text{||}v_{t}\text{|}{\text{|}}_{\sum_{t}^{-1}}^{2}\\ \;s.t.\;X_{t}^{2}=F^{2}(X_{t-1}), \end{array}\;(12)$$

where *X _t_* ∼ *N*( *X _t_*; ${\hat{X}}_{t}$ , *P _t_*) are state distributions for each time step and ${\hat{X}}_{1:N_{T}}$ is the collection of all these state distributions.

### 2.3 Derivation of optimization-based recursive Bayesian estimator

Since we are interested in online estimation with a low computational cost, a consistent recursive estimator of ( 12) is derived in the following. It estimates the states for the current time step and reduces to the Extended Kalman Filter (EKF) as one special case. For the derivation, we rewrite the general model Equation (9) to Equation (11) as


$$\;X_{t}^{1}=F_{t}^{1}(X_{t-1})+w_{t},\;(13)$$

$$\;X_{t}^{2}=\underset{=F_{t}^{2}(X_{t-1})}{\underset{︸}{\underset{\varepsilon_{t}\to 0}{\lim}{\tilde{F}}_{t}^{2}(X_{t-1},\;\varepsilon_{t}),}}\;(14)$$

$$\;Y_{t}=H_{t}(X_{t})+v_{t},\;(15)$$

with *w _t_* ∼ *N*(0, $Q_{t}^{1}$ ) and *ε _t_* ∼ *N*(0, $Q_{t}^{2}$ ). ${\tilde{F}}_{t}^{2}$ is a slightly modified version of $F_{t}^{2}$ , where small artificial noise ( *ε _t_*) is added to the variables $X_{t-1}^{2}$ that emerge in $F_{t}^{2}$ . Let the complete state $X_{t}={\left({\left(X_{t}^{1}\right)}^{T},{\left(X_{t}^{2}\right)}^{T}\right)}^{T}\in {\mathbb{R}}^{N_{1}+N_{2}}$ consist of *X* ^1^ ∈ ℝ ^*N*_1_^ and *X* ^2^ ∈ ℝ ^*N*_2_^. We can now linearize Equation (14) with respect to the noise at *ε _t_* = 0 to obtain


$$\;X_{t}^{1}=F_{t}^{1}(X_{t-1})+w_{t},\;(16)$$

$$\;X_{t}^{2}={\tilde{F}}_{t}^{2}(X_{t-1},\;0)+\underset{\varepsilon_{t}\to 0}{\lim}\underset{={\tilde{Q}}_{t}^{2}}{\underset{︸}{W_{t}Q_{t}^{2}W_{t}^{T}}},\;(17)$$

$$\;Y_{t}=H_{t}(X_{t})+v_{t},\;(18)$$

for $W_{t}=\frac{\partial {\tilde{F}}^{2}(X_{t-1},0)\;}{\partial \varepsilon_{t}}$ with the property lim _*ε _t_* →0_ $Q_{t}^{2}$ = 0 _*N*_2_× *N*_2__ i.e. $Q_{t}^{2}$ converges to the zero matrix for decreasing *ε _t_*. Without loss of generality, we assume ${\tilde{Q}}_{t}^{2}$ to be invertible for all *ε _t_* > 0 (otherwise replace ${\tilde{Q}}_{t}^{2}$ with ${\tilde{Q}}_{t}^{2}$ + *ε*_*t*_ *I*_*N*_2_× *N*_2__). Analogously to the derivation of the (E)KF in 30, we can write Equation (16) to Equation (18) as linear equation system for time steps *t* = 1 . . . *k*, with measurements up to time step *m* ≤ *k*, and with the collective state vector *X*_1: *k*_ = ( $X_{1}^{T}$ ,…, $X_{k}^{T}$ ) ^*T*^ (corresponds to *z _k_* in 30). The least squares solution to this equation system is the minimizing solution to the following objective function


$$J_{k\text{|}m}(X_{1:k})=\frac{1}{2}\text{||}X_{1}-{\hat{X}}_{1}\text{|}{\text{|}}_{P_{1}^{-1}}^{2}+\underset{=\lim_{\varepsilon_{t}\to 0}\;\frac{1}{2}\sum_{t=2}^{k}\text{||}X_{t}-F_{t}(X_{t-1})\text{|}{\text{|}}_{Q_{t}^{-1}}^{2}}{\underset{︸}{\frac{1}{2}\sum_{t=2}^{k}\text{||}X_{t}^{1}-F_{t}^{1}(X_{t-1})\text{|}{\text{|}}_{{\left(Q_{t}^{1}\right)}^{-1}}^{2}+\underset{\varepsilon_{t}\to 0}{\lim}\frac{1}{2}\sum_{t=2}^{k}\text{||}X_{t}^{2}-F_{t}^{2}(X_{t-1})\text{|}{\text{|}}_{{\left({\tilde{Q}}_{t}^{2}\right)}^{-1}}^{2}}}+\frac{1}{2}\sum_{t=1}^{m}\text{||}Y_{t}-H_{t}(X_{t})\text{|}{\text{|}}_{\sum_{t}^{-1}}^{2},\;(19)$$

with


$$\;F_{t}(X_{t-1})=\left(\begin{array}{c} F_{t}^{1}(X_{t-1})\\ F_{t}^{2}(X_{t-1}) \end{array}\right),\;\;Q_{t}=\left(\begin{array}{cc} Q_{t}^{1} & 0\\ 0 & Q_{t}^{2} \end{array}\right).\;(20)$$

In ( 19), *J*_*k*| *m*_( *X*_1: *k*_) describes the objective with motion model residuals up to time step *k* and measurement residuals up to time step *m* and thus state estimates up to time step *k*.

Note that the limes of *ε* must not be taken in ( 19), since this would lead to a singular matrix for the residual weighting of the third term. Instead, a recursive estimation form is derived in the following. This allows taking the limes in the final form and naturally leads to a consistent prediction. To obtain a recursive formulation, similarly to 30, we proceed by considering the case *k* = *m*, where there are measurements available for each time step.

The prediction step consists of determining the estimate ${\hat{X}}_{k|k-1}$ based on ${\hat{X}}_{k-1|k-1}$ ∼ *N*( *X*_*k*−1| *k*−1_; ${\hat{X}}_{k-1|k-1}$ , *P*_*k*−1| *k*−1_), the latter including the measurements up to time step *k* − 1. To derive the prediction step, we write the objective ( 19) in recursive form as


$$\;J_{k\text{|}k-1}(X_{1:k})=J_{k-1\text{|}k-1}(X_{1:k-1})+\underset{\varepsilon_{k}\to 0}{\lim}\frac{1}{2}\text{||}X_{k}-F_{k}(X_{k-1})\text{|}{\text{|}}_{{\left(Q_{k}\right)}^{-1}}^{2}.\;(21)$$

Here, *J*_*k*−1| *k*−1_( *X*_1: *k*−1_) relates to the prior distribution *p*( *X*_*k*−1_| *Y*_1: *k*−1_) ≈ *N*( *X*_*k*−1_; ${\hat{X}}_{k-1|k-1}$ , *P*_*k*−1| *k*−1_) and, exploiting the Markov assumption, could be represented as


$$\;J_{k-1\text{|}k-1}(X_{1:k-1})\underset{\text{Markov}}{\underset{︸}{\;=\;}}J_{k-1\text{|}k-1}(X_{k-1})=\text{||}X_{k-1\text{|}k-1}-{\hat{X}}_{k-1\text{|}k-1}\text{|}{\text{|}}_{P_{k-1\text{|}k-1}^{-1}}^{2}.\;(22)$$

The gradient of ( 21) is


$$\;\nabla J_{k\text{|}k-1}(X_{1:k})\underset{\text{Markov}}{\underset{︸}{\;=\;}}\nabla J_{k\text{|}k-1}(X_{k-1},X_{k})=\frac{\partial J_{k\text{|}k-1}(X_{1:k})}{\partial (X_{k-1},X_{k})}\;(23)$$

$$\;=\underset{\varepsilon_{k}\to 0}{\lim}\left(\begin{array}{c} \nabla J_{k-1\text{|}k-1}(X_{k-1})+\nabla F_{k}{\left(X_{k-1}\right)}^{T}Q_{k}^{-1}(X_{k}-F_{k}(X_{k-1}))\\ Q_{k}^{-1}(X_{k}-F_{k}(X_{k-1})) \end{array}\right),\;(24)$$

with ∇ *J*_*k*−1| *k*−1_( *X*_*k*−1_) = $\frac{\partial J_{k-1|k-1}(X_{k-1})}{\partial X_{k-1}}$ and ∇ *F _k_* ( *X*_*k*−1_) = $\frac{\partial F_{k}(X_{k-1})}{\partial X_{k-1}}$ .

The Hessian of ( 21) is


$$\;\nabla^{2}J_{k\text{|}k-1}(X_{k-1},X_{k})=\underset{\varepsilon_{k}\to 0}{\lim}\left(\begin{array}{cc} \nabla^{2}J_{k-1|k-1}(X_{k-1})+G(X_{k-1},X_{k},\varepsilon_{k}) & \nabla F_{k}{\left(X_{k-1}\right)}^{T}Q_{k}^{-1}\\ Q_{k}^{-1}\nabla F_{k}{\left(X_{k-1}\right)}^{T} & Q_{k}^{-1} \end{array}\right),\;(25)$$

with


$$\;G(X_{k-1},X_{k},\varepsilon_{k})=\nabla^{2}F_{k}(X_{k-1})Q_{k}^{-1}(X_{k}-F_{k}(X_{k-1}))+\nabla F_{k}{\left(X_{k-1}\right)}^{T}Q_{k}^{-1}\nabla F_{k}(X_{k-1}).$$

Since ( 21) is a quadratic objective function and *∇* ^2^ *J*_*k*| *k*−1_( *X*_*k*−1_, *X _k_*) is positive definite, a single iteration of Newton’s method yields the minimum. Thus, the prediction can be written as


$$\;\left(\begin{array}{c} {\hat{X}}_{k-1}\\ {\hat{X}}_{k} \end{array}\right)=\left(\begin{array}{c} X_{k-1}\\ X_{k} \end{array}\right)-\nabla^{2}J_{k\text{|}k-1}{\left(X_{k-1},X_{k}\right)}^{-1}\nabla J_{k\text{|}k-1}(X_{k-1},X_{k}),\;(26)$$

for any initial guess for $\left(\begin{array}{c} X_{k-1}\\ X_{k} \end{array}\right)$ .

Now, observing that if the estimate of the previous time step was a minimum $\nabla J_{k-1|k-1}\left({\hat{X}}_{k-1}\right)=0$ and, since choosing ${\hat{X}}_{k}=F_{k}\left({\hat{X}}_{k-1}\right)$ sets all other terms in ( 24) to zero, the initial guess $\left(\begin{array}{c} {\hat{X}}_{k-1}\\ F_{k}\left({\hat{X}}_{k-1}\right) \end{array}\right)$ yields a minimum of ( 21). Considering this in ( 26), i.e. that the second term on the right-hand side equals zero, the estimate is this initial guess and the prediction mean reads


$$\;{\hat{X}}_{k-1\text{|}k}={\hat{X}}_{k}=F_{k}({\hat{X}}_{k-1}).\;(27)$$

The prediction covariance can now be computed by evaluating the Hessian ( 25) at the prediction mean $\left(\begin{array}{c} {\hat{X}}_{k-1}\\ F_{k}\left({\hat{X}}_{k-1}\right) \end{array}\right)$ , which gives


$$\;\begin{array}{l} \;\;\nabla^{2}J_{k\text{|}k-1}({\hat{X}}_{k-1},{\hat{X}}_{k})=\\ \underset{\varepsilon_{k}\to 0}{\lim}\left(\begin{array}{cc} \nabla^{2}J_{k-1|k-1}({\hat{X}}_{k-1})+\nabla F_{k}{\left({\hat{X}}_{k-1}\right)}^{T}Q_{k}^{-1}\nabla F_{k}({\hat{X}}_{k-1}) & \nabla F_{k}{\left({\hat{X}}_{k-1}\right)}^{T}Q_{k}^{-1}\\ Q_{k}^{-1}\nabla F_{k}{\left({\hat{X}}_{k-1}\right)}^{T} & Q_{k}^{-1} \end{array}\right). \end{array}\;(28)$$

With the inverse of Schur’s complement the inverse of ( 28) can be written as


$$\;\begin{array}{l} \;\;\nabla^{2}J_{k\text{|}k-1}^{-1}({\hat{X}}_{k-1},{\hat{X}}_{k})=\\ \underset{\varepsilon_{k}\to 0}{\lim}\left(\begin{array}{cc} \nabla^{2}J_{k-1|k-1}^{-1}({\hat{X}}_{k-1}) & \nabla^{2}J_{k-1|k-1}^{-1}({\hat{X}}_{k-1})\nabla F_{k}{\left({\hat{X}}_{k-1}\right)}^{T}\\ \nabla F_{k}({\hat{X}}_{k-1})\nabla^{2}J_{k-1|k-1}^{-1}({\hat{X}}_{k-1}) & Q_{k}+\nabla F_{k}({\hat{X}}_{k-1})\underset{=P_{k-1|k-1}}{\underset{︸}{\nabla^{2}J_{k-1|k-1}^{-1}({\hat{X}}_{k-1})}}\nabla F_{k}{\left({\hat{X}}_{k-1}\right)}^{T} \end{array}\right). \end{array}\;(29)$$

Since the inverse Hessian ( 29) is computed for both time steps, we can extract the covariance for the predicted mean ( 27) from the lower right corner of ( 29). Therefore,


$$\;P_{k\text{|}k-1}=\underset{\varepsilon_{k}\to 0}{\lim}Q_{k}+\nabla F_{k}({\hat{X}}_{k-1})P_{k-1\text{|}k-1}\nabla F_{k}{\left({\hat{X}}_{k-1}\right)}^{T}.\;(30)$$

In this form *Q _k_* does not need to be inverted and can be singular, thus we can now take the limes *ε _k_* → 0 to obtain


$$\;P_{k\text{|}k-1}=Q_{k}+\nabla F_{k}({\hat{X}}_{k-1})P_{k-1\text{|}k-1}\nabla F_{k}{\left({\hat{X}}_{k-1}\right)}^{T}\;(31)$$

with


$$Q_{k}=\left(\begin{array}{cc} Q_{k}^{1} & 0\\ 0 & 0 \end{array}\right).$$

Thus, a well defined, consistent and recursive prediction for the model ( 9) and ( 10) is given via the mean ( 27) and the covariance ( 31). With this we have derived the prediction


$$\;p(X_{k}\text{|}Y_{1:k-1})\approx N(X_{k};{\hat{X}}_{k\text{|}k-1},P_{k\text{|}k-1}).\;(32)$$

To obtain the posterior distribution


$$p(X_{k}\text{|}Y_{1:k})\approx N(X_{k};{\hat{X}}_{k\text{|}k},P_{k\text{|}k}),$$

we have to include the measurement update for time step *k*. From the distributional point of view the posterior is proportional to the product of the prediction with the likelihood


$$p(X_{k}\text{|}Y_{1:k})\propto p(Y_{k}\text{|}X_{k})p(X_{k}\text{|}Y_{1:k-1}).$$

From the objective function point of view we have to approximate


$$\;J_{k\text{|}k}(X_{k})=\underset{\text{likelihood}}{\underset{︸}{\text{||}Y_{k}-H_{k}(X_{k})\text{|}{\text{|}}_{\sum_{k}^{-1}}^{2}}}+\underset{\text{prediction}}{\underset{︸}{J_{k\text{|}k-1}(X_{k-1:k})}}.\;(33)$$

This can be rewritten as


$$\;J_{k\text{|}k}(X_{k})=\text{||}Y_{k}-H_{k}(X_{k})\text{|}{\text{|}}_{\sum_{k}^{-1}}^{2}+\text{||}X_{k}-{\hat{X}}_{k\text{|}k-1}\text{|}{\text{|}}_{P_{k\text{|}k-1}^{-1}}^{2},\;(34)$$

where the prediction yields a prior on the states for the measurement update. With the non-linear least squares problem ( 34), and the exact prediction ( 27) and ( 31), we can derive multiple EKF variants, like the Iterated EKF (via the Gauss-Newton method) or a Levenberg-Marquardt Iterated EKF, depending on the numerical optimization scheme used to minimize this objective. This is, in detail, described in 27 and can be analogously applied here. In this work, we use a Gauss-Newton method with line-search for the measurement models to obtain the estimate


$$\;{\hat{X}}_{k\text{|}k}=\underset{X_{k}}{\min}J_{k\text{|}k}(X_{k}).\;(35)$$

The covariance is computed as


$$\;P_{k\text{|}k}=(I-KH)P_{k\text{|}k-1},\;(36)$$

$$\;H={\frac{\partial H_{k}(s)}{\partial s}|}_{s={\hat{X}}_{k\text{|}k-1}},\;(37)$$

$$\;K=P_{k\text{|}k-1}H^{T}{\left(HP_{k\text{|}k-1}H^{T}+\sum_{k}\right)}^{-1},\;(38)$$

with *P*_*k*| *k*−1_ from ( 31).

### 2.4 Motion models

This section specifies the equations for constructing *F _t_* in ( 9) and ( 10). The IMU motion models assume constant acceleration and constant angular velocity from time *t* − 1 to *t*, with sampling time *∆t*, hence


$$\;I_{i,t}^{N}=I_{i,t-1}^{N}+\text{Δ}t{\dot{I}}_{i,t-1}^{N}+\frac{{\left(\text{Δ}t\right)}^{2}}{2}\;{\ddot{I}}_{i,t-1}^{N}\;(39)$$

$$\;{\dot{I}}_{i,t}^{N}={\dot{I}}_{i,t-1}^{N}+\text{Δ}t\;{\ddot{I}}_{i,t-1}^{N}\;(40)$$

$$\;{\ddot{I}}_{i,t}^{N}={\ddot{I}}_{i,t-1}^{N}+\text{Δ}t\;w_{t}^{a}\;(41)$$

$$\;\chi_{t}^{NI_{i}}=\chi \;\left(q_{t-1}^{NI_{i}}⊙\exp\left(\frac{\text{Δ}t}{2}\omega_{I_{i,}t-1}^{NI_{i}}\right)\right)\;(42)$$

$$\;\omega_{I_{i,t}}^{NI_{i}}=\omega_{I_{i},t-1}^{NI_{i}}+\text{Δ}t\;w_{t}^{\omega },\;(43)$$

where $w_{t}^{a}∼N\left(0,Q_{t}^{a}\right),w_{t}^{\omega }∼N\left(0,Q_{t}^{\omega }\right)$ denote independent normally distributed process noises, ⊙ denotes the quaternion multiplication and exp denotes the quaternion exponential. The transition models for the joint ( *i*, *j*) ∈ and fixed point *l* ∈ ℐ parameters are


$$\;\;{\left\{J_{i,j,t}^{I_{p}}=J_{i,j,t-1}^{I_{p}}\right\}}_{p\in \{i,j\}}\;(44)$$

$$\;\;J_{\text{fix},t}^{I_{l}}=J_{\text{fix},t-1}^{I_{l}}.\;(45)$$

### 2.5 Measurement models

This section presents the different measurement models used for constructing *H _t_* in ( 11). These comprise models for exploiting the actual sensor measurements, i.e. the IMU data, as well as, models to incorporate assumptions concerning the kinematic chain (connected joints) and its interaction with the environment (fixed position, zero velocity detections). The latter are required for positional drift compensation. In this work, we evaluate two optional measurement models for positional drift compensation: 1) fixed position (assuming that one IMU moves around a fixed point in the navigation frame) is evaluated on the simulated three link chain (see Section 3.2), 2) zero velocity detections are evaluated on the lower body walking data from humans (see Section 3.3) and a humanoid robot (see Section 3.4). Note, for the purpose of positional drift compensation, position or velocity based information needs to be induced into the estimation at least for one IMU, since the joint connection models (( 49, 50) below) ensure the propagation of such induced information through the kinematic chain. Moreover, the magnetometer measurement model is also optional, and evaluation results are presented with and without using this model.

***2.5.1 IMU data.*** Let *g ^N^* be the gravity vector in the navigation frame, the accelerometer and gyroscope measurement models are ^13^


$$\;\;y_{a,t}^{I_{i}}={\left(R_{i,t}^{NI}\right)}^{T}\;\left({\ddot{I}}_{i}^{N}-g^{N}\right)+v_{t}^{a}\;(46)$$

$$\;y_{\omega ,t}^{I_{i}}=\omega_{I_{i},t}^{NI_{i}}+v_{t}^{\omega },\;(47)$$

where $v_{t}^{a}∼N\left(0,\sum_{t}^{a}\right),v_{t}^{\omega }∼N\left(0,\sum_{t}^{\omega }\right)$ denote independent normally distributed measurement noises. To omit heading drift, the magnetometer measurements can be used. The following (implicit) model is constructed to only provide information in the heading direction and omit information about the local dip angle, which is a common way to reduce the influence of magnetic disturbances ^31^:


$$\;0=\text{atan}2\left[\frac{{\left(R_{t}^{NI_{i}}y_{m,t}^{I_{i}}\right)}_{y}}{{\left(R_{t}^{NI_{i}}y_{m,t}^{I_{i}}\right)}_{x}}\right]+v_{t}^{\text{mag}}\;(48)$$

where (·) _*x*_, (·) _*y*_ denote the *x* and *y* component of the respective vector and $v_{t}^{mag}∼N\left(0,\sum_{t}^{\text{mag}}\right)$ denotes normally distributed measurement noises.

***2.5.2 Joint connections.*** The skeleton structure, i.e. the fact that the segments of the tracked skeleton (kinematic chain) are connected by joints, is also incorporated via two measurement models. The information is operationalized by equating the two IMU-centered position states per joint in the navigation frame, as well as, the joint velocities:


$$\;{\left\{0=I_{i,t}^{N}+R_{t}^{NI_{i}}J_{i,j,t}^{I_{i}}\;-\;\left(I_{j,t}^{N}+R_{t}^{NI_{j}}J_{i,j,t}^{I_{j}}\right)+v_{t}^{\text{cp}}\right\}}_{\left(i,j\right)\in J}\;(49)$$

$$\;\;{\left\{0={\dot{I}}_{i,t}^{N}+R_{t}^{NI_{i}}\left(\left[\omega_{I_{i},t}^{NI_{i}}\times \right]J_{t}^{I_{i}}\right)\;-\;\left({\dot{I}}_{j,t}^{N}+R_{t}^{NI_{i}}\left(\left[\omega_{I_{j},t}^{NI_{j}}\times \right]J_{t}^{I_{j}}\right)\right)+v_{t}^{\text{cv}}\right\}}_{\left(i,j\right)\in J},\;(50)$$

where [ *a*×] denotes the cross-product matrix of vector *a* and $v_{t}^{\text{cp}}∼N\left(0,\sum_{t}^{\text{cp}}\right),v_{t}^{\text{cv}}∼N\left(0,\sum_{t}^{\text{cv}}\right)$ denote independent normally distributed measurement noises. Note, from a theoretical point of view, the joint velocity constraint could be left out, since IMU position and velocity are completely linked through zero process noise in the time update. However, the experimental evaluation on the three-link chain ( Section 3.2) supports using both models. Moreover, note that the above measurement models represent time integrations of the acceleration-level joint connection measurement model proposed in 26. Comparative results are also provided in the experimental evaluation.

***2.5.3 Fixed positions.*** This model assumes an IMU *i* moving around a fixed point $P_{\text{fix}}^{N}$ in the navigation frame (e.g. the upper arm IMU moving around the shoulder joint in the simulated three-link chain). The fixed point is then estimated in the respective IMU coordinate system *I _i_* as additional IMU-centered joint position state $J_{\text{fix},t}^{I_{i}}$ . The associated measurement model is:


$$\;0=P_{\text{fix}}^{N}\;-(I_{i,t}^{N}+R_{t}^{NI_{i}}\;J_{\text{fix},t}^{I_{i}})+v_{t}^{\text{fix}},\;(51)$$

where $v_{t}^{\text{fix}}∼N\left(0,\sum_{t}^{\text{fix}}\right)$ denotes normally distributed measurement noise.

***2.5.4 Zero velocity detections.*** For all IMUs *c* ∈ ᑕ *ℐ*, zero velocity *m*_*c*, *t*_ = 1, *m _c,t_* ∈ {0, 1} is detected, based on a sliding window of inertial measurements $y_{a,t-b:t}^{I_{c}}\;\text{and}\;y_{\omega ,t-b:t}^{I_{c}}$ , using the stance hypothesis optimal (SHOE) detector ^32^. Note, in case of multiple detections, only the IMU with the lowest SHOE value is kept. The measurement model for a zero velocity detection ( *m*_*c*, *t*_ = 1) is 


$$\;0={\dot{I}}_{c,t}^{N}+v_{t}^{\text{zvd}}\;,\;(52)$$

where $v_{t}^{\text{cvd}}∼N\left(0,\sum_{t}^{\text{cvd}}\right)$ denotes normally distributed measurement noise. Note, in a locomotion scenario, zero velocities are typically regularly detected at the foot IMUs during the stance phases. In locomotion activities with flight phases, such as running or jumping, these events become less frequent, and the estimation can show drift, if there is no other drift compensating mechanism.

### 2.6 State initialization

There exist different approaches for state initialization in the context of IHMC, e.g. initially assumed poses ^33, 34^ or orientation initializations *R ^NI _i_^*, *i* ∈ *ℐ*, while the other quantities are assumed to be zero ^35^.

In this work, we also assume the kinematic chain to be initially stationary, i.e. IMU states ${\dot{I}}_{i,0}^{N},{\ddot{I}}_{i,0}^{N},\omega_{I_{i},0}^{NI},\forall i$ are initialized with $N\left(0,\sum_{init}^{v}\right),N\left(0,\sum_{init}^{a}\right),N\left(0,\sum_{init}^{\omega }\right)$ . Moreover, the IMU positions $I_{i,0}^{N}$ are also initialized with $N\left(0,\sum_{init}^{p}\right),\forall i.$

The IMU orientations $\chi_{0}^{NI_{i}},\forall i$ are initialized using the accelerometer and the magnetometer measurements via the triad method ^36^ as e.g. in 13. Note, this is the only point in time, where magnetometer measurements are required.

In order to support Monte Carlo simulations in the evaluation, the joint positions are initialized randomly with $J_{i,j,0}^{I_{i}},J_{i,j,0}^{I_{j}}∼N\left(0,\sum_{init}^{J}\right)$ , with $\sum_{init}^{J}=l^{2}I_{3\times 3}$ and *l* is the maximal segment length in order to obtain a reasonable distribution ( *l* = 0.4 in the experiments). The fixed positions are treated analogously.

### 2.7 On observability of the estimated quantities

Following the notation about structural and practical observability, based on 37, we can identify the following structural non-observabilities. The Gauge freedom ^20, 38^ in initial position $I_{i,0}^{N},\forall i$ and initial velocity ${\dot{I}}_{i,0}^{N},\forall i$ (before zero velocity updates) are correct only up to additive offsets, since no absolute references exist for these estimates. To mitigate this problem, we define prior distributions for the initial position and velocity, with the assumptions stated in Section 2.6.

Regarding practical observability, the joint position estimates need "sufficient" excitation to be observable. Ideally, all degrees of freedom of the relative rotations between the neighboring IMUs have to be excited. This can be analyzed using an approach similar to 26. Alternatively, we propose a scalar joint position uncertainty measure based on the covariance of our state estimator. This can be exploited to indicate sufficient motion excitation for a given joint (e.g. by applying a threshold), or, in other words, as convergence indicator for joint position estimation. Let $\sum \left(J_{i,j,t}^{I}\right)$ be the average of the marginalised (3 × 3) covariance matrices of joint ( *i*, *j*) as estimated in both IMU coordinate systems *I _i_*, *I _j_*. We then calculate the scaled largest eigenvalue of this matrix with


$$\;s_{J_{i,j,t}^{I}}^{max}=s_{c}\sqrt{{\text{EV}}_{\sum (J_{i,j,t}^{I})}^{max}},\;(53)$$

where *s _c_* ≈ 3.37 corresponds to the square root of the 99%-quantile of the Chi-squared distribution with three degrees of freedom. This approximates the calculation of the 99% credibility region using a scaled spectral norm. Respective evaluations for different movement scenarios can be found in Section 3.

While, in this work, magnetometers are always used to initialize the IMU orientations via the triad method (see Section 2.6), the magnetometer measurement model ( 48) can be used to obtain observable orientation estimates with respect to the heading direction in the navigation frame. However, if only relative rotations are relevant, and sufficient motion is present to allow for local observability at each joint, as formalized in 39, the magnetometer measurements can be neglected during tracking. According to 39, local non-observability is given, if acceleration and jerk at a joint are co-linear. This only happens in case of no motion or motion along the gravitational vector. In Section 3, we also evaluate the effects of using or omitting magnetometer measurements during tracking.

## 3 Evaluation and results

This section presents the evaluation of the proposed approach with respect to different estimated quantities (IMU orientations, joint positions) and aspects (convergence speed, accuracy, drift behaviour). The evaluation metrics are summarized in Section 3.1. Evaluation is performed on three different motion scenarios: simulated data of a three-link kinematic chain fixed in space (sinusoidal motion in all degrees of freedom) ( Section 3.2), real lower body human gait data from 25 healthy persons measured at two points in time ( Section 3.3), and gait data captured from a humanoid robot ( Section 3.4). The covariance settings used in the proposed recursive Bayesian estimator are listed in Table 1 (in the order they are introduced in Section 2.4 to Section 2.6). Sensor biases were subtracted from the real IMU data as pre-processing step.

**Table 1.** Estimator covariances used in the experimental evaluation.

Covariance	Value
*Q ^*a*^*	3 · 10 ^5^ · *I*_3×3_
*Q ^*ω*^*	10 ^4^ · *I*_3×3_
Σ ^*a*^	10 ^–2^ · *I*_3×3_
Σ ^*ω*^	10 ^–3^ · *I*_3×3_
Σ ^*mag*^	10 ^–2^ · *I*_3×3_
Σ ^*cp*^	10 ^–4^ · *I*_3×3_
Σ ^*cv*^	10 ^–3^ · *I*_3×3_
Σ ^*fix*^	10 ^–4^ · *I*_3×3_
Σ ^*cvd*^	10 ^–4^ · *I*_3×3_
$\sum_{init}^{v}$	1 · *I*_3×3_
$\sum_{init}^{a}$	1 · *I*_3×3_
$\sum_{init}^{\omega }$	1 · *I*_3×3_
$\sum_{init}^{\chi }$	10 ^–6^ · *I*_3×3_
$\sum_{init}^{p}$	1 · *I*_3×3_
$\sum_{init}^{J}$	1.6 · 10 ^–1^ · *I*_3×3_

### 3.1 Evaluation metrics

The joint position estimates are evaluated via the segment length errors. Let *sl _i_* be the length of segment *S _i_* with corresponding IMU *I _i_* and connecting joints *J*_*h*, *i*_ and *J*_*i*, *j*_. The estimated segment length at time *t* is then


$$\;sl_{i,t}=\text{||}J_{i,j,t}^{I_{i}}-J_{h,i,t}^{I_{i}}\text{|}{\text{|}}_{2},\;(54)$$

and the error can be calculated with respect to the reference *sl*_*i*, *ref*_ as


$$\;E_{i,t}^{sl}=\text{||}sl_{i,t}-sl_{i,ref}\text{|}{\text{|}}_{2}.\;(55)$$

The reference is either chosen as the ground truth length of the segment (simulation study, Section 3.2), the estimated segment length at the end of a reference motion (gait study, Section 3.3), or a measured reference (robot, Section 3.4). In the second case, the reliability of the joint position estimates is additionally evaluated exploiting gait data from the same person measured at two different days, with different IMU mountings. In the first case (simulation study), we also evaluate the end-point errors for each estimated joint position in each IMU coordinate system, e.g.


$$\;E_{i,j,t}^{J}=\text{||}J_{i,j,t}^{I_{i}}-J_{i,j,ref}^{I_{i}}\text{|}{\text{|}}_{2},\;(56)$$

where reference joint position $J_{i,j,ref}^{I_{i}}$ is taken from the constructed model.

The estimated relative IMU orientations $R_{t}^{I_{i}I_{j}}=R_{t}^{I_{i}N}R_{t}^{NI_{j}},\forall \left(i,j\right)$ are evaluated on the angle errors


$$\;E_{i,j,t}^{rel}=\text{|}\phi (R_{t}^{I_{i}I_{j}}\;R_{ref,t}^{I_{j}I_{i}})\text{|},\;(57)$$

where $\phi \left(R\right)=\text{arccos}\left(\frac{\text{trace}\left(R\right)-1}{2}\right)$ ^13^. Analogously, the global IMU orientations are evaluated in the simulation study. To summarize the errors (deviations from the references) over a complete sequence in one number, the root mean squared error (RMSE) is exploited as


$$\;E_{X}^{RMSE}=\frac{1}{N_{T}}\sqrt{\sum_{t=1}^{N_{T}}\sum_{l\in X}{\left(E_{l,t}\right)}^{2}},\;(58)$$

where *X* is the index set of the respective measure, e.g. *l* = ( *i*, *j*) ∈ for joint ( *i*, *j*) and *E* denotes an error, e.g. *E _J_* or *E ^rel^* as defined above.

To evaluate the convergence behaviour of the joint position estimates, we also consider the sample standard deviation (RMSE Std.) of the RMSEs for different initial values obtained via Monte Carlo sampling.

The validity of the relative IMU orientations is furthermore evaluated via the coefficient of determination *R* ^2^ ^40^. Moreover, systematic biases and drift in IMU orientation estimates are evaluated via the ordinary least product regression (OLP) coefficients ^41^.

### 3.2 Simulation study on a three-link chain

This section presents the evaluation results of the proposed approach using the fixed position measurement model for positional drift compensation (see Section 2.5.3) on simulated (noisy) IMU data. The results regard the observability of the joint positions as well as the validity of the IMU orientation estimates.

***3.2.1 Experimental setup.*** Tests are performed on simulated IMU data from a three-link chain fixed in space, as illustrated in Figure 2. The skeleton consists of three segments *S _i_*, *i* = 0, 1, 2 with IMUs *I _i_*, *i* = 0, 1, 2. The I2S calibrations assumed for simulation are:


$$\;T^{S_{0}I_{0}}=\left(\begin{array}{cccc} 0 & 0 & 1 & 0.1\\ 0 & 1 & 0 & 0\\ -1 & 0 & 0 & 0.15\\ 0 & 0 & 0 & 1 \end{array}\right),\;T^{S_{1}I_{1}}=\left(\begin{array}{cccc} 0 & -1 & 0 & 0\\ 0 & 0 & 1 & 0.1\\ -1 & 0 & 0 & 0.2\\ 0 & 0 & 0 & 1 \end{array}\right),\;T^{S_{2}I_{2}}=\left(\begin{array}{cccc} 0 & -1 & 0 & 0\\ 0 & 0 & 1 & 0.1\\ -1 & 0 & 0 & 0.05\\ 0 & 0 & 0 & 1 \end{array}\right).\;(59)$$

The first segment is fixed in space at the starting point and the other two segments are attached via two three-dimensional rotary joints.

The motion and IMU data simulation are taken from 13 (Section 2.6.3, *sim-fast-artificial* sequence). In short, the motion is obtained by assuming sinusoidal angle profiles for all joint degrees of freedom. This satisfies the need for sufficient motion for the observability of the joint positions. Based on the above, the IMU data, ground truth IMU poses and joint positions are calculated.

The average measured execution time per timestep (for three IMUs) was 1.38 ms when running our single-threaded unoptimized C++ implementation on an octa-core AMD Ryzen 7 7800X3D (Base Clock: 4.2 GHz).

***3.2.2 Joint position estimates.*** The convergence speed of the joint position estimation is evaluated with *N* = 10 Monte Carlo samples for the initial joint positions as well as for the noises applied during IMU data simulation. Comparable results were obtained with and without magnetometer model.

**Figure 3.**
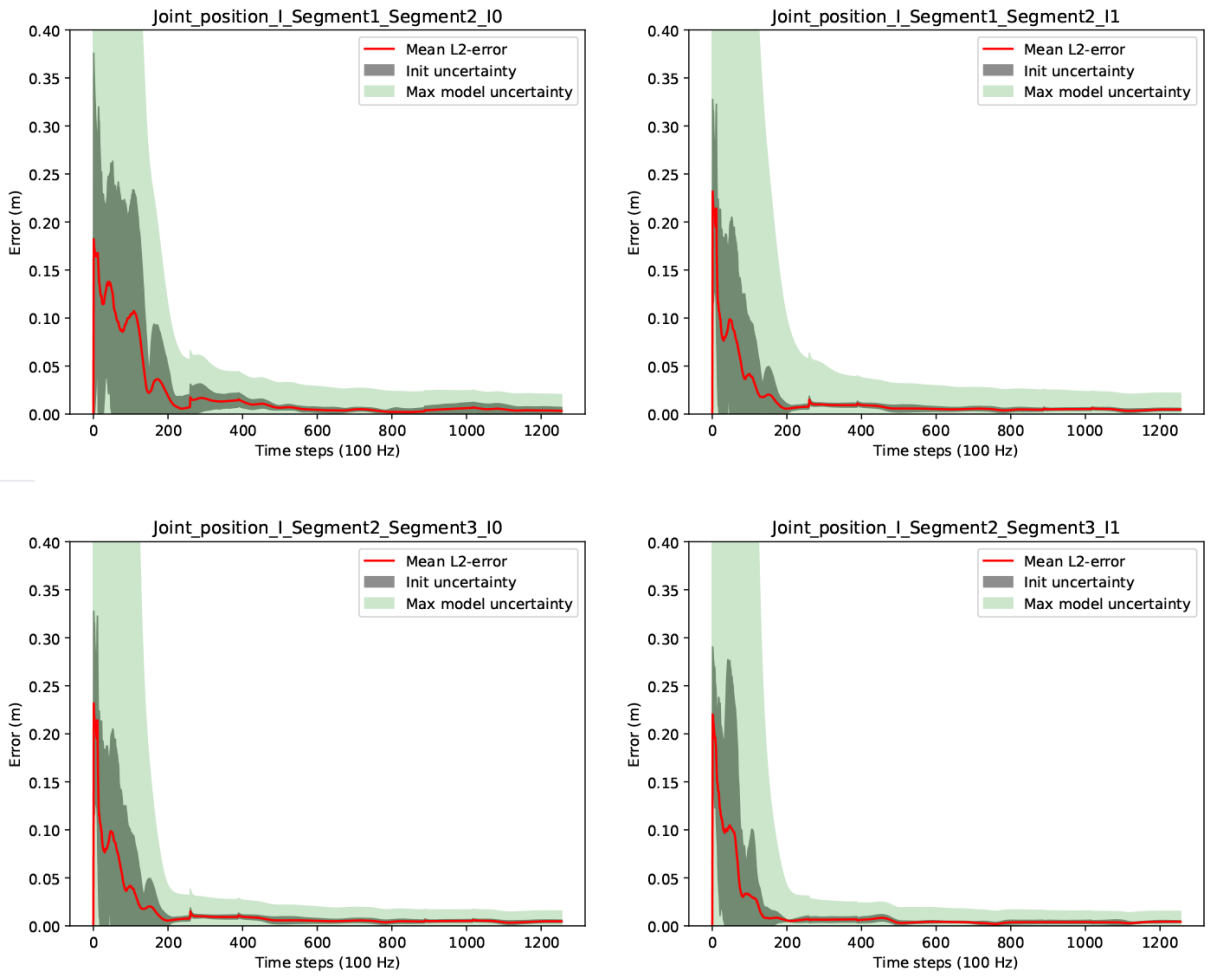
Three-link chain: Joint position estimation errors and uncertainties over two motion cycles, i.e. 1256 time steps at 100 Hz.

Figure 3 shows the convergences of the joint position estimates. In the figure, the red lines correspond to the end-point errors ( 56) for each estimated joint position in each IMU coordinate system. The dark grey shaded areas (labelled init uncertainty) correspond to the sample standard deviations over the errors due to different joint initializations from the Monte Carlo samples, and the light green shaded areas (labelled max model uncertainty) correspond to the maximum estimated joint position uncertainties ( 53) over the Monte Carlo samples. The latter is our currently proposed scalar joint position uncertainty measure to indicate the motion excitation for the respective joint. Note, during online estimation, we have no Monte Carlo samples, and the uncertainty is based on the estimation covariance.

It can be observed that the joint position estimates are converged with very low init uncertainty after about 200 samples, which corresponds to about 2 seconds of data. Given the Monte Carlo sampling, this can be considered a robust result for random initializations for the current experimental setup. It is also visible, that the proposed joint position uncertainty measure (max model uncertainty) is a valid convergence indicator in this experiment (the light green shaded areas include the red lines). Comparable results were obtained for the estimation of the fixed position.

**Figure 4.**
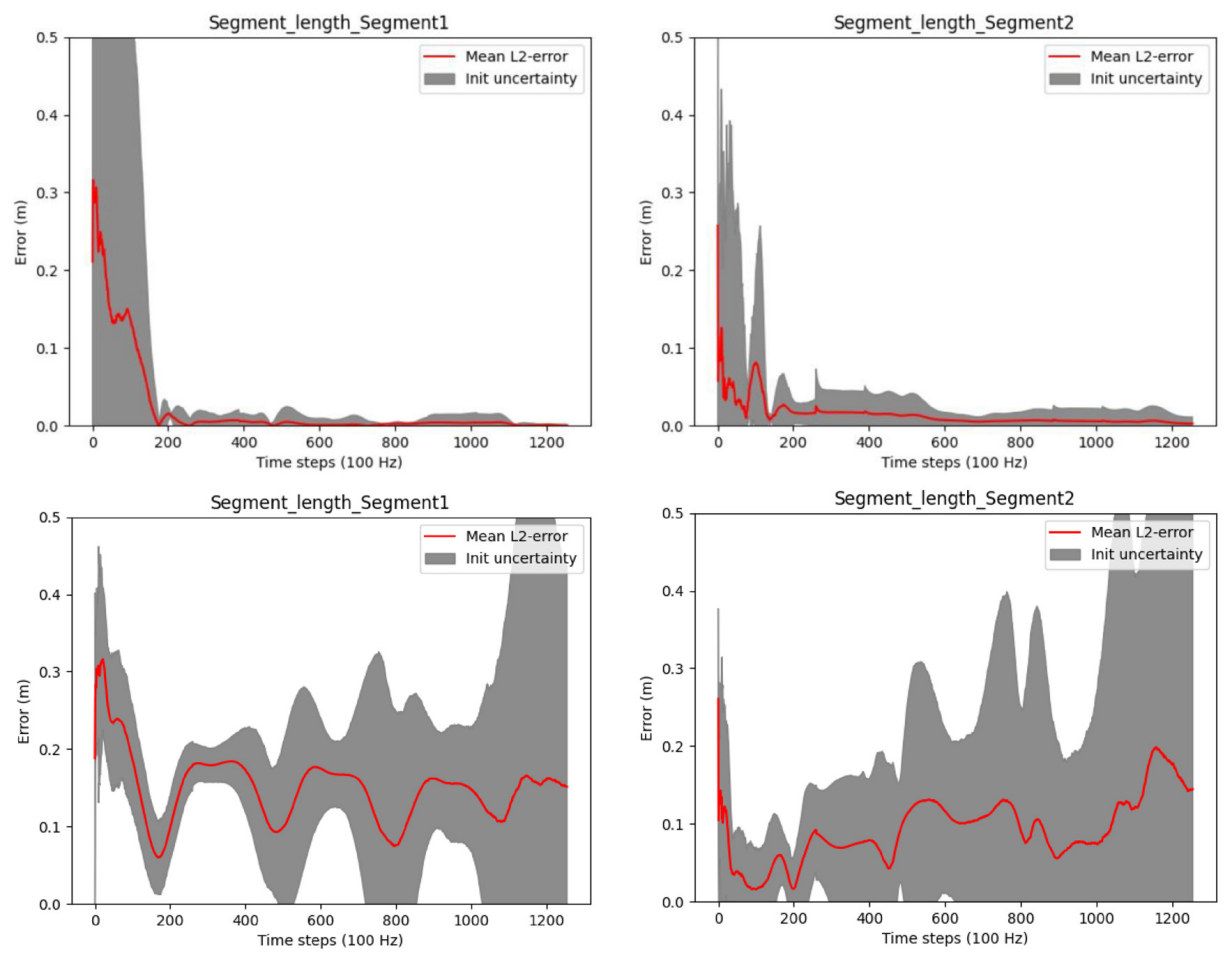
Three-link chain: Segment length estimation errors and uncertainties over two motion cycles, i.e. 1256 time steps at 100 Hz. The upper row shows the results when using our proposed method (joint connection measurement models on position and velocity level). The lower row shows the results when using only an acceleration-level joint connection measurement model.

Figure 4 shows results concerning the segment length errors as calculated via ( 55). Note, since the segment length computation requires the joint positions, only two lengths can be calculated. For the third (distal) segment of the three link chain, where no fixation in space is assumed, the segment length calculation is not possible. In the figure, the upper row shows the results from our proposed online method operating on a complete kinematic chain and using position- and velocity-level joint connection measurement models. With this, the final absolute segment length errors (after time step 1256) are very low with 1.1 millimeters for the first and 1.5 millimeters for the second segment. As mentioned in Section 2.5.2, a previous offline and per-joint method for joint position estimation employed an acceleration-level joint connection measurement model ^26^. For comparison, we tested a variant of our proposed method, where an acceleration-level joint connection measurement model was used instead of the position- and velocity-level models. The latter is based on Equation (9) in 26, whereas the angular accelerations were calculated using a difference quotient on the angular velocities in the state, and the measurement model uncertainty was set to *I*_3×3_. The results in the lower row of Figure 4 show no convergence and significantly higher errors and uncertainties compared to our proposed method. This is due to the missing drift correction on IMU positions and velocities over the complete kinematic chain, when purely using the acceleration-level measurement model. Note, segment lengths are calculated based on multiple IMU poses in our method. We also evaluated a variant using only the position-level connection measurement model (omitting the velocity-level model), which resulted in slightly lower accuracy and slower convergence. However, using the acceleration-level model in addition to the other two, did not improve the results but increase the computational cost. The following experiments only consider our proposed method (using position- and velocity-level joint connection measurement model).

**Figure 5.**
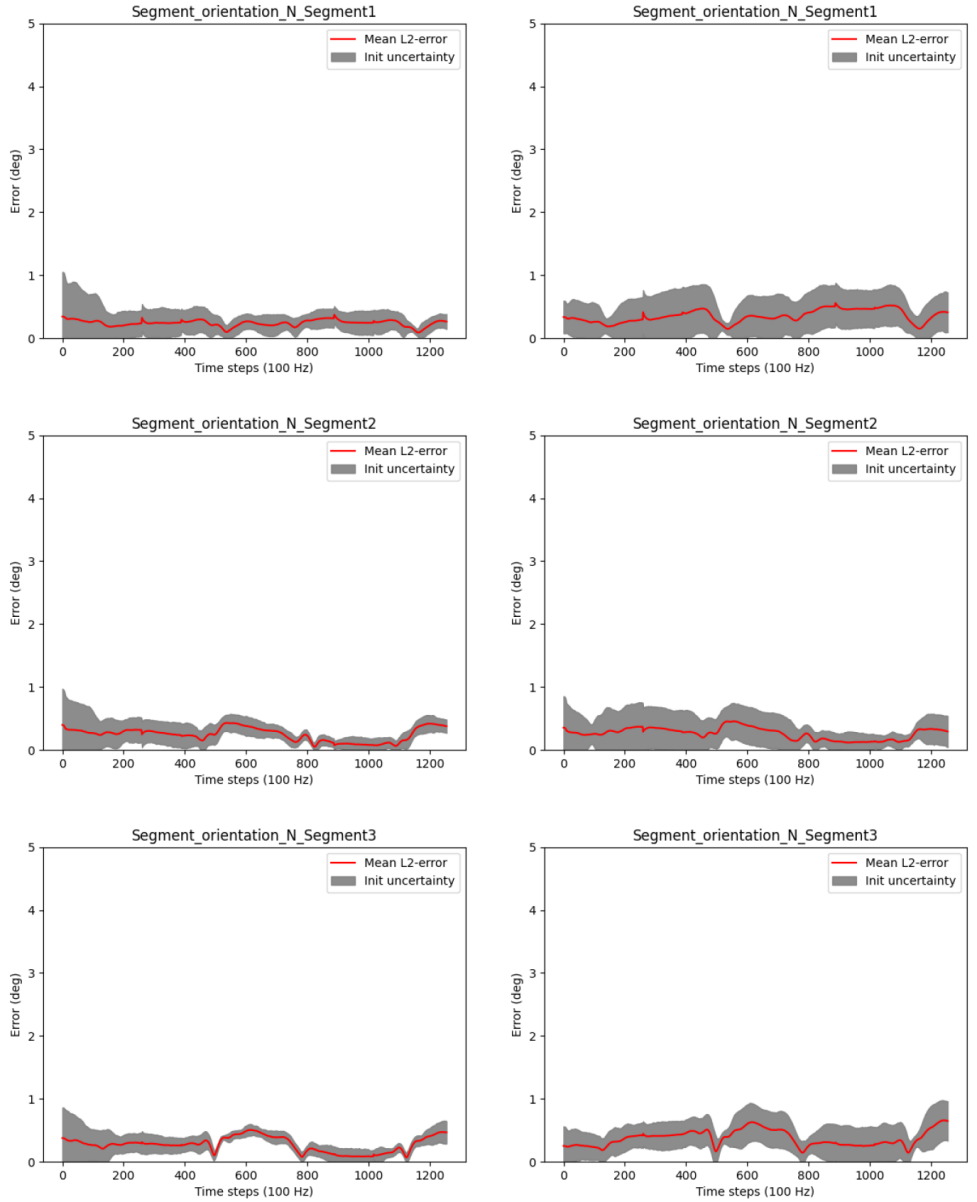
Three-link chain: Angle errors and init uncertainties of estimated global IMU orientations with magnetometers (left) and without magnetometers (right) for randomly initialized joint positions.

***3.2.3 IMU orientation estimates.*** To assess the IMU orientation estimates and the influence of the joint position estimation on these with and without magnetometers, we also investigated the angle errors of the estimated relative and global IMU orientations ( 57) for the randomly initialized joint positions. The results for the global IMU orientations are shown in Figure 5. The results for the relative IMU orientations look similar and are therefore omitted here.

The figure shows that the IMU orientation estimates with or without magnetometers, are not significantly affected by the joint position estimation. The errors stay mostly below 1°, independently of joint position initialization and magnetometer usage. This holds also for the relative IMU orientations. Slightly higher init uncertainties can be observed without the magnetometer model, which is expected due to the lower amount of information fed to the estimator when omitting magnetometer information. Note that, even if not visible in this experimental setting, without magnetometers, the global heading direction is not observable, which might result in drift in the heading direction when processing longer data sequences with other disturbances (besides noise), see e.g. 33. This is, however, not the focus of the present work.

***3.2.4 Long term estimation.*** To assess the long term stability of the proposed approach after convergence, we investigated the relative and global IMU orientation estimates over 300 motion cycles (i.e. *N* = 300 · 629 samples at 100 Hz, which corresponds to 31.45 minutes) with respect to systematic errors. For this, the joint positions and initial IMU poses were initialized correctly. Moreover, the relative IMU orientation estimates are without the magnetometer model, while the global IMU orientation estimates are with the magnetometer model (since the global heading direction is otherwise not observable, as pointed out in the previous section). As introduced in Section 3.1, we computed the OLP coefficients, the *R* ^2^ scores and the RMSEs between the estimated orientations and the ground truth orientations, both reduced to single angles ( 57), considering all samples. The results show that the proposed approach performs very well in all measures: The absolute additive OLP coefficients are all far below 1° (0.11° on average over all joints and segments) indicating only very minor biases, whereas the OLP scaling factors are all 1.0 indicating no systematic error like drift. These results are confirmed by the *R* ^2^ scores (all equal to 1.0) and the very low RMSEs all far below 1° (0.20° on average over all joints and segments). These results demonstrate the consistency and long term stability of the proposed approach for the noisy simulated IMU data in a scenario with sufficient motion excitation.

### 3.3 Human lower body scenario

This section presents the evaluation results of the proposed approach using the zero velocity detections measurement model for positional drift compensation (see Section 2.5.4) on real lower body human gait data, namely the TUK-6-minute-walking dataset ^42^, first introduced in 33.

**Figure 6.**
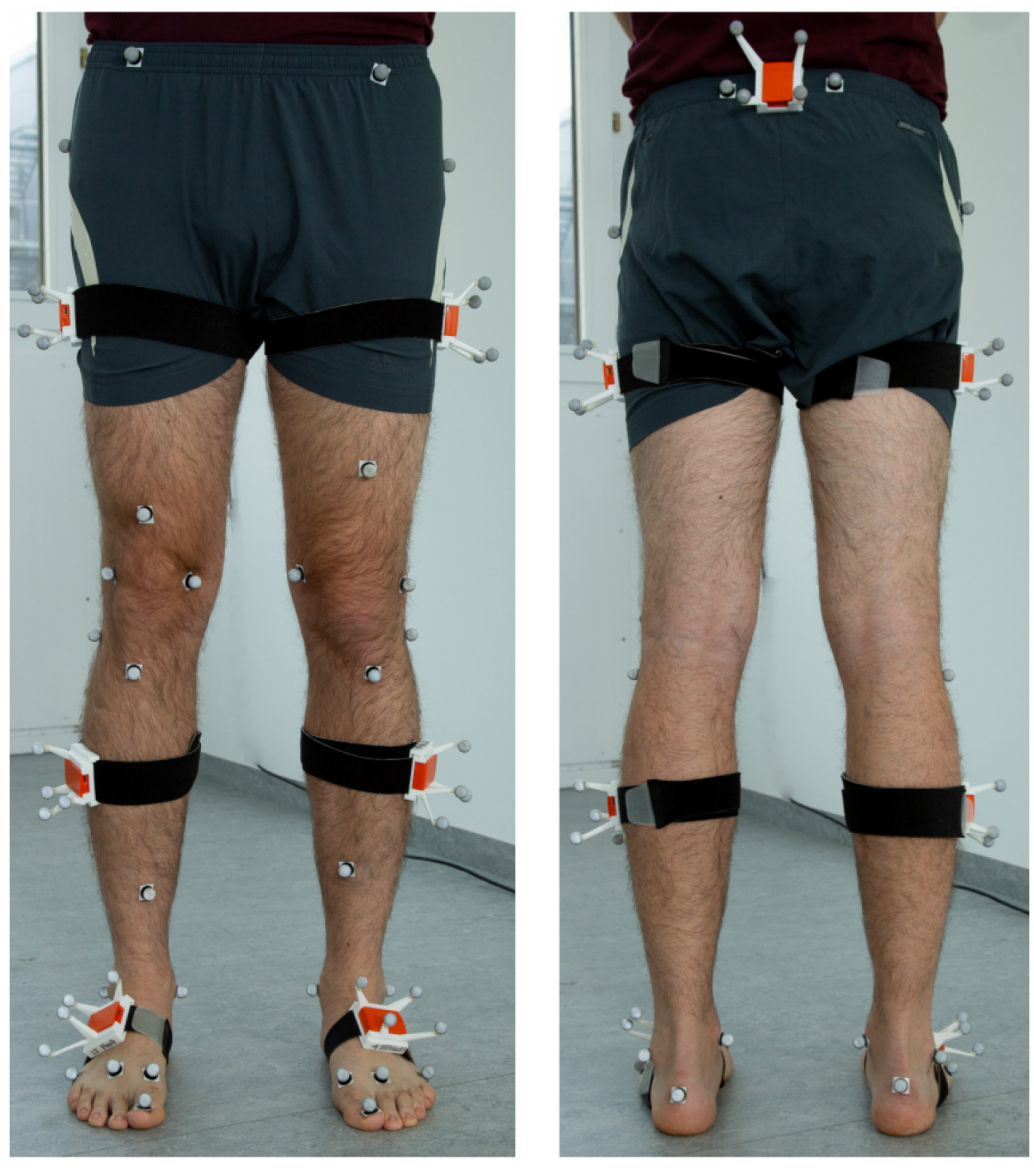
Experimental setup of the TUK-6-minute-walking dataset with IMUs and optical markers.

***3.3.1 Experimental setup.*** The experimental setup is shown in Figure 6. The dataset contains synchronized lower body IMU and optical reference data from 25 young, healthy test persons (15 females, age 24.60 ± 2.70 years; 69.44 ± 12.17 kg and 1.75 ± 0.08 m in height) and two sessions (6.75 ± 2.26 days between test and retest). The hardware setup consisted of twelve OptiTrack Prime 13 cameras (NaturalPoint, Inc., Corvallis, OR, USA) and seven XSens MTw Awinda (Xsens Technologies BV, Enschede, The Netherlands) IMUs. The study was approved by the ethical committee of the Rheinland-Pfälzische Technische Universität (RPTU) (date of approval 5.12.2017), formerly known as Technische Universität Kaiserslautern (TUK) and meets the criteria of the declaration of Helsinki. After receiving all relevant study information, the participants signed an informed consent to the study including a permission to publish the data.

In contrast to the previously described simulated motion, there is no exact ground truth data available for the lower body dataset. In particular, the joint position reconstruction from skin markers typically involves approximations, such as regression models ^43^. Therefore, we focus our evaluation of the joint position estimates on test-retest reliability of the derived segment lengths (rather than joint position validity). For the IMU orientations, we focus on validity (comparison with the optical data), since the optical marker bodies rigidly attached to the IMUs can be very accurately tracked to deduce the IMU orientations. The subsequent results are all without the magnetometer model. Comparable results were obtained with the magnetometer model.

The average measured execution time per timestep (for seven IMUs) was 15.3 ms when running our single-threaded unoptimized C++ implementation on an octa-core AMD Ryzen 7 7800X3D (Base Clock: 4.2 GHz).

**Figure 7.**
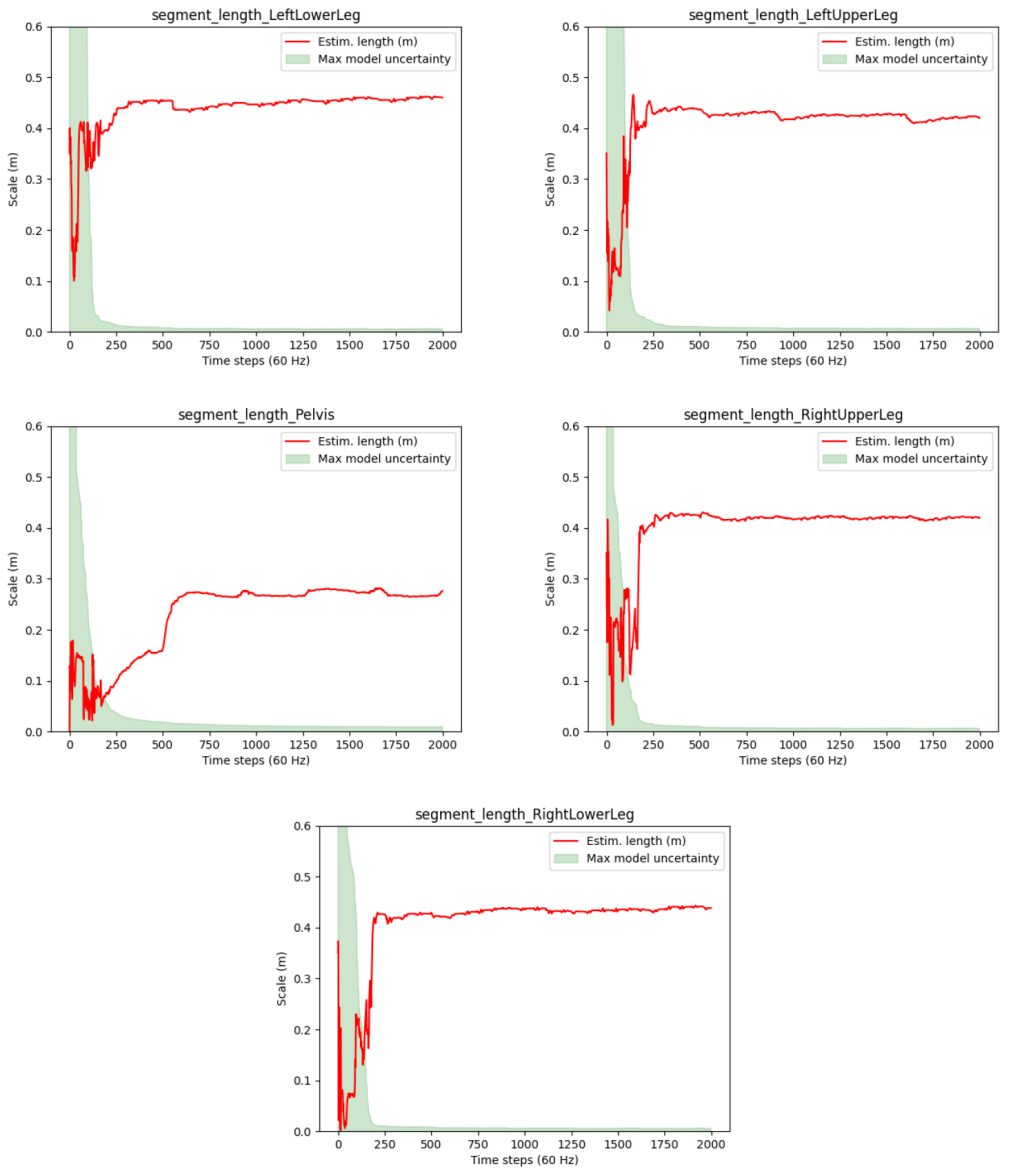
Human lower body: Estimated segment lengths and uncertainties for a selected test person (P1) of the TUK-6-minute-walking dataset.

***3.3.2 Joint position estimates.*** The convergence speed of the joint position estimation is exemplified in Figure 7 for one test person via the segment lengths derived from the joint position estimates (red lines). The meaning of the shaded areas corresponds to the description in Section 3.2.2. However, note that the red lines indicate the segment lengths rather than the estimation errors and that the max model uncertainty reflects the maximum uncertainty of the two joint positions.

It can be observed that the segment lengths (and hence the joint position estimates) reach steady states after about 300 samples, which corresponds to about 5 seconds of the 60 Hz data. Moreover, the estimated lengths seem feasible for a human body. Also the drops in the proposed joint position uncertainty measure (max model uncertainty) roughly correspond with reaching the steady states (given that the max model uncertainty is associated to only one of the joint positions). However, the red lines keep showing visible swings between frames 200 and 300. A deeper analysis showed that sudden changes in the estimates correlate with the zero velocity detections, which might be inaccurate, but at the same time significantly reduce the model uncertainties.

For quantifying the reliability, we computed statistical differences in segment length estimates after 2000 samples between test and retest data. The assumption is, that the segment lengths (per person) do not change between test and retest, where the same person is measured with the same hardware performing the same walking activity on two different days. However, note that the IMU mountings, which heavily influence the estimated quantities in the local IMU coordinate systems, are different between test and retest, though they were not explicitly varied. The I2S differences between test and retest (approximated from the optical data) are quantified in Table 2. Table 3 shows the rather small RMSEs (below 2 centimeters) over all persons of the final segment length estimates between test and retest and the sample standard deviations due to Monte Carlo sampling (init uncertainties).

**Table 2.** Human lower body: Mean and maximum I2S position and orientation (angle) differences over all segments and all test persons between test and retest in the TUK-6-minute-walking dataset.

I2S difference	Mean	Maximum
*I ^S^* [m]	0.0192	0.0716
*Φ* ( *R ^SI^*) [°]	7.4206	88.2683

**Table 3.** Human lower body: Reliability of the estimated segment lengths on the TUK-6-minute-walking dataset.

Segment name	RMSE [m]	Init uncertainty
LeftLowerLeg	0.00160116	3.29466e-08
LeftUpperLeg	0.01426190	6.05016e-07
Pelvis	0.01485470	3.99128e-07
RightLowerLeg	0.00907722	1.66203e-08
RightUpperLeg	0.0187878	3.46729e-07

Altogether, it can be concluded that straight walking provides sufficient motion excitation for obtaining reasonable joint position estimates via the proposed approach, though motion excitation is significantly lower in the frontal and transversal plane compared to the sagittal plane.

**Figure 8.**
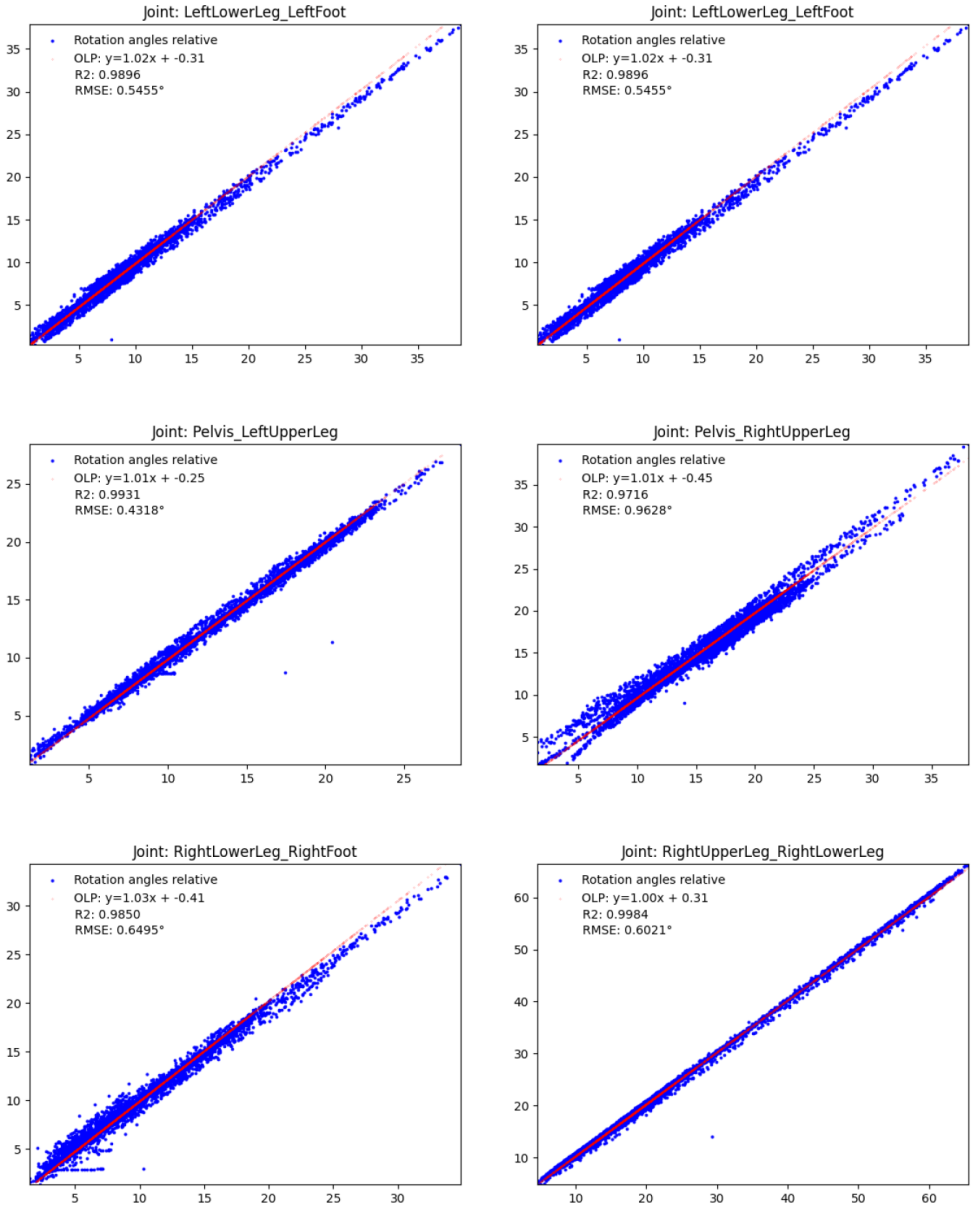
Human lower body: Validity of the estimated IMU orientations on the TUK-6-minute-walking dataset (without magnetometer information): estimated joint angles (y-axis) vs. joint angles obtained from the optical data (x-axis), with OLP coefficients, RMSEs and *R* ^2^ scores. In the plots, the orange dots represent the OLP coefficients and the blue dots represent the individual angle pairs (estimated over optical reference). A perfect estimation results in blue dots on the line through the orange dots.

***3.3.3 IMU orientation estimates.*** To evaluate the validity of the relative IMU orientation estimates, we computed the OLP coefficients, the *R* ^2^ scores and the RMSEs between the estimated orientations and the orientations obtained from the optical data during the test session, as before, both reduced to single angles and considering all samples. The results are shown in Figure 8. The red OLP regression lines correlate well with the blue dots (indicating the individual angle samples), though the latter show higher variances compared to the results from the three-link chain. In detail, the absolute additive OLP coefficients are all far below 1° indicating only minor biases, whereas the OLP scaling factors are all close to 1.0 indicating no systematic errors such as drift. These results are confirmed by the *R* ^2^ scores (all close to 1.0) and the low RMSEs (all below 1°). The detailed numbers are given in the figure.

### 3.4 Humanoid robot lower body scenario

This section presents the evaluation results of the proposed approach using the zero velocity detections measurement model for positional drift compensation (see Section 2.5.4) on real lower body data captured from a humanoid robot ^44^.

**Figure 9.**
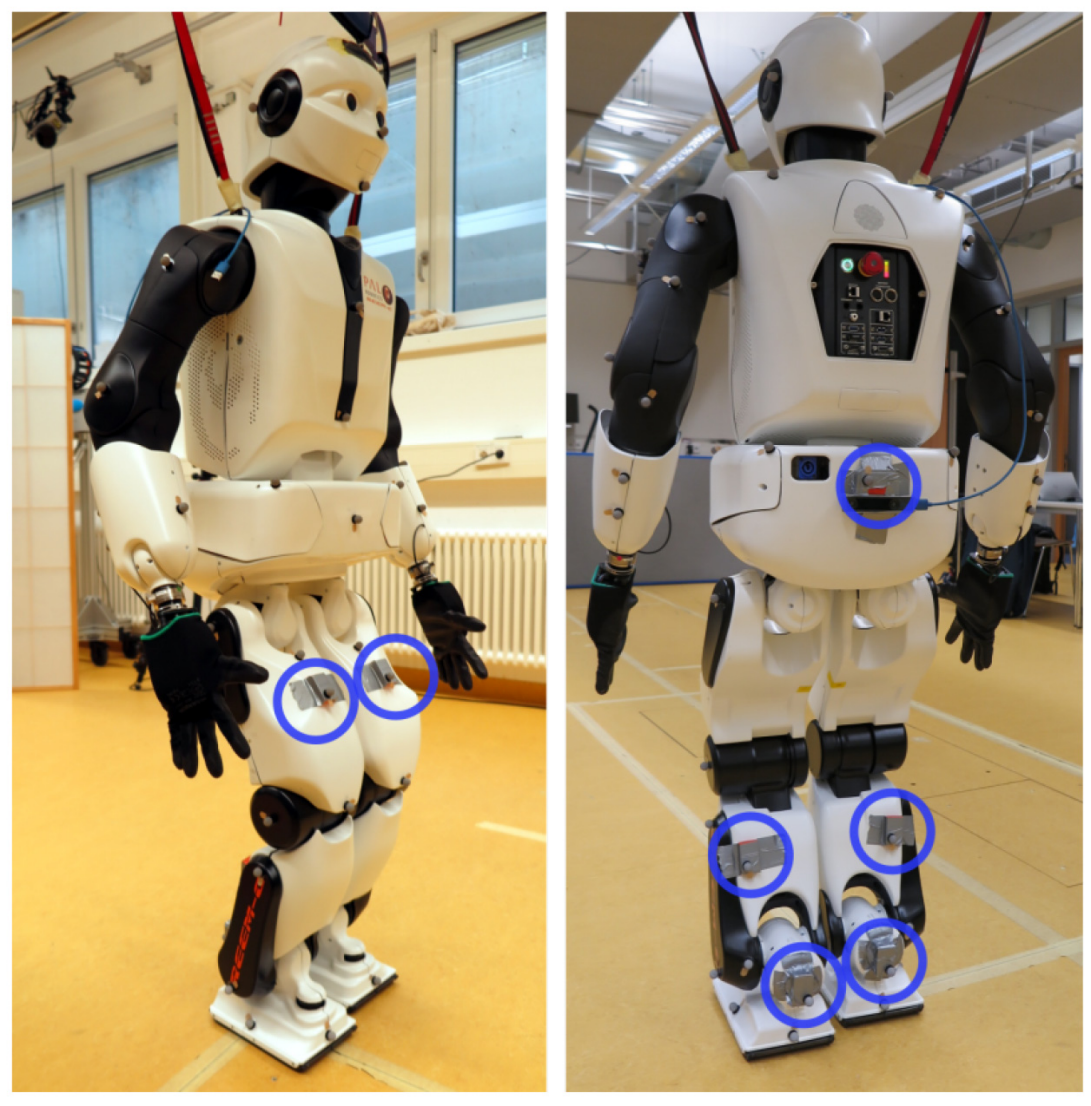
Humanoid robot Reem-C from Pal Robotics (Barcelona, Spain) equipped with optical markers and IMUs. The location of the IMUs are highlighted with blue circles.

***3.4.1 Experimental setup.*** The same IMU hardware and a comparable IMU setup were used as in the previous experiment. In particular, seven IMUs were attached on the foot, lower leg, upper leg and pelvis segments of the humanoid robot Reem-C from Pal Robotics (Barcelona, Spain). IMU data was collected at 100 Hz. Moreover, the robot motion was synchronously captured with a marker-based optical system (Qualisys AB, Göteborg, Sweden) at 150 Hz. The setup is shown in Figure 9. The captured activity consists of the robot walking in a circle along the edge of the recording volume of the optical motion capture system. The considered IMU data sequence has 40.000 samples corresponding to about 6.67 minutes.

The Reem-C robot is equipped with electrically driven joints. The ankles have two degrees of freedom, the knees are hinge joints (one degree of freedom) and the hips are ball-and-socket joints (three degrees of freedom). Compared to the previous experiment with humans, this setup has some advantages but also challenges with respect to the evaluation of the proposed approach. Advantages are that the robot has rigid segments which do not induce soft tissue artefacts, that it has precisely known segment lengths and that the mechanical joints allow rotations around fixed and predefined axes.

According to the manual, lower and upper legs have each a length of 0.3 meters, and the pelvis has a width (distance from hip joint center to hip joint center) of 0.15 meters.

Potential challenges are the very low acceleration and velocity of the robot motion, given that the joint position estimation requires sufficient motion excitation for convergence (see Section 2.7), and the hinge joints (knees). In case of a perfect hinge joint, the relative rotation between the two associated segments only takes place around a fixed axis, which results in the fact that the joint position estimates are not identifiable, since each position on the hinge axis represents a valid solution to the models in 2.5.2 ^18, 20, 45^. The following results are only without magnetometer model.

**Figure 10.**
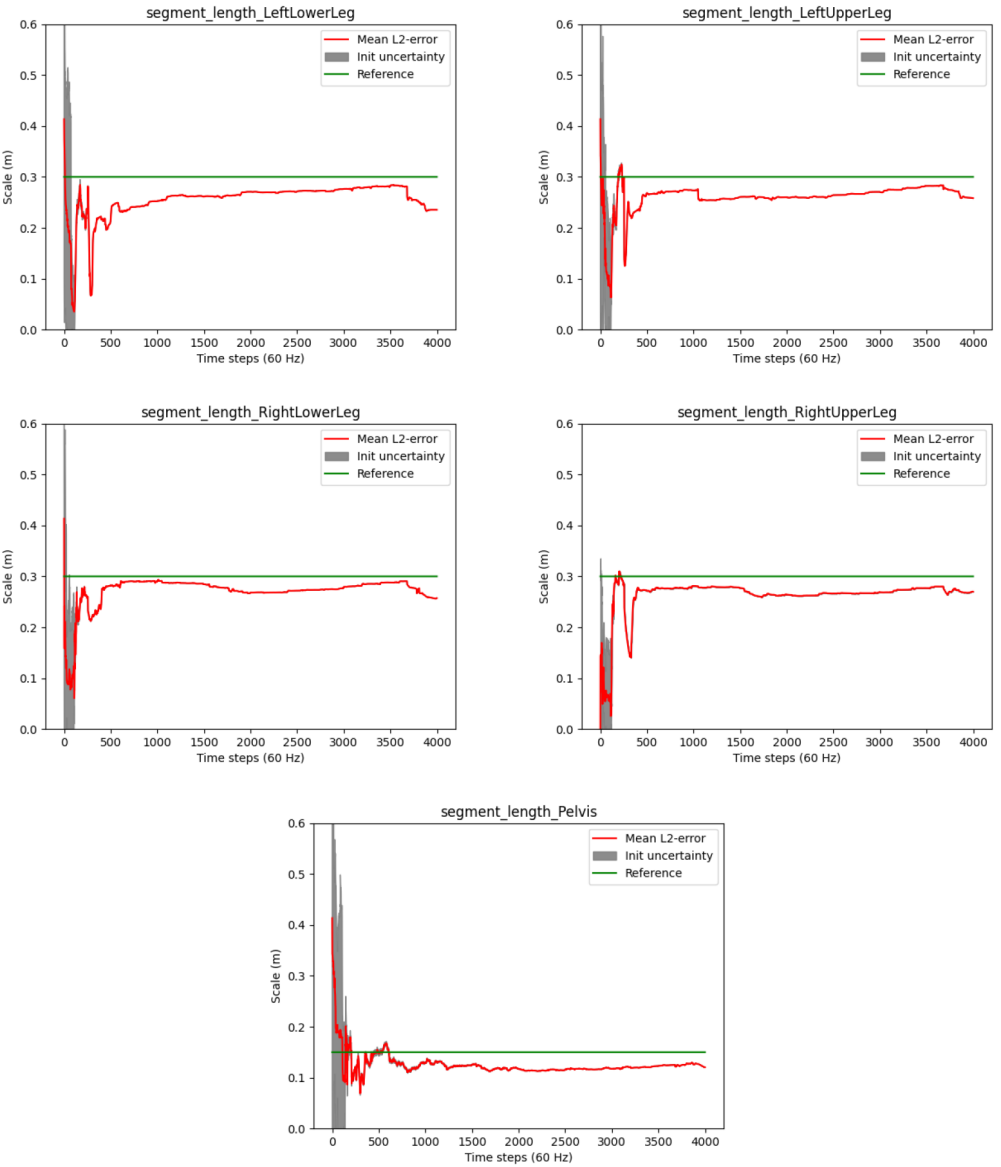
Humanoid robot: Estimated segment lengths (red lines) and init uncertainties in comparison to the ground truth lengths (green lines).

***3.4.2 Joint position estimates.*** The convergences of the segment length estimates are illustrated in Figure 10. The plots show slower convergences as compared to the human walking experiment (cf. Figure 7). Table 4 shows the accuracy of the estimated segment lengths with respect to the above mentioned reference measures, with the RMSEs and standard deviations over the last 100 samples of the motion sequence. The segment length estimate at the pelvis is most accurate with a RMSE of about 1.5 centimeters. This might be due to the higher motion variability at the hip joints. The RMSEs of the other segments range between 1.5 and 3.7 centimeters. Altogether, reasonably stable estimates close to the ground truth measurements are reached for all segments despite the overall slow motion and the hinge joints of the robot.

**Table 4.** Humanoid robot: Accuracy of the estimated segment lengths (without magnetometer model).

Segment name	RMSE [m]	Std.
LeftLowerLeg	0.0333680	1.00993e-05
LeftUpperLeg	0.0157264	5.00482e-06
Pelvis	0.0147733	2.14394e-05
RightLowerLeg	0.0355856	1.30683e-05
RightUpperLeg	0.0368549	6.38446e-06

However, note the occasionally appearing abrupt changes in the segment length estimates in Figure 10, e.g. around frame 1000 at the left upper leg. A deeper analysis showed that these result from false positive zero velocity detections (due to the slow robot motion), where erroneous information is fed into the estimator via the model described in Section 2.5.4. Similar artefacts were observed in the previous experiment, so that is seems desirable to decouple the joint position estimation from the global positional drift reduction mechanism.

***3.4.3 IMU orientation estimates.*** The accuracy of the relative IMU orientations with respect to the optical reference, reduced to single angles, is shown in Table 5. Altogether, the RMS angle errors are very low (below 2°).

**Table 5.** Humanoid robot: Accuracy of the relative IMU orientation estimates (without magnetometer model).

Joint name	RMSE [°]	Std.
LeftFoot_LeftLowerLeg	0.855757	0.382451
LeftLowerLeg_LeftUpperLeg	1.981640	0.654190
LeftUpperLeg_Pelvis	1.362980	0.600270
Pelvis_RightUpperLeg	0.586756	0.344031
RightFoot_RightLowerLeg	1.186340	0.646329
RightLowerLeg_RightUpperLeg	0.847667	0.514045

## 4 Conclusions and open challenges

This section summarizes and draws conclusions from the evaluation in Section 3 and then outlines limitations, open challenges and future work.

### 4.1 Summary and conclusions

In this work we derived and evaluated an optimization-based recursive Bayesian estimation approach for simultaneous estimation of IMU poses (orientation and position) and joint positions for kinematic chains of different configurations. To the best of our knowledge, this is the first real-time capable approach for simultaneous IMU pose and joint (and fixed point) position estimation. The approach is recursive and uses only the data of the current timestep. Moreover, it was shown to run in real-time (below IMU framerate) in the experimental evaluation (15.3 ms average measured processing time per timestep on 60 Hz data from seven IMUs). Note that the used C++ implementation is single-threaded and not optimized; e.g. it uses numerical derivatives and no sparse matrices. We also propose an indicator for joint or fixed point position estimation convergence ( 53). We evaluated the proposed approach in different experimental scenarios: simulated IMU data from an arm-like three-link kinematic chain fixed in space as well as human and robotic lower body walking data.

The proposed estimation approach performs excellently on noisy simulated IMU data from a three link chain with a fixed position and with sufficient motion in all degrees of freedom. This concerns the convergence speed (below 200 samples at 100 Hz) and accuracy of the joint positions (final segment length estimation error below 1.5 millimeters) as well as the accuracy of the IMU orientations (all errors below 1°), even without magnetometer usage and during a longer data sequence (over 30 minutes). This demonstrates the overall consistency of the derived estimator.

Also on real IMU data from different locomotion scenarios with zero velocity detections, very good results were obtained for the accuracy of the magnetometer-free relative IMU orientation estimates (RMSE below 2°). Moreover, reasonable results were obtained for the joint position estimates in terms of convergence speed (about 300 samples at 60 Hz for the humans and between 500 and 1000 samples at 100 Hz for the robot), reliability (RMSE of segment length estimates below 2 centimeters between test and retest for the humans) and accuracy (RMSE of segment length estimates below 4 centimeters for the robot). This demonstrates the successful application of the proposed approach on real data and in scenarios with sub-optimal conditions, such as the comparably planar nature of walking movements in general, in particular for the robot with perfect hinges at the knees, in combination with a very slow walking speed. Altogether, the proposed approach is a promising method for real-time IMU pose and joint position estimation without the need for I2S calibration. Hence, in this sense, the approach is calibration-free. Note that a pose based I2S calibration procedure (e.g. as in 46) could of course be used, with the additional advantage that the I2S position can be directly computed from the joint position estimates, as mentioned in Section 2.1.

Casting this in the direction of visual human pose tracking, our approach allows for IMU mounting independent estimation of joint positions in a navigation frame. Hence, it paves the way for the effortless estimation of segment lengths (in physical units), as well as the integration with joint position estimates from computer vision systems.

### 4.2 Limitations, open challenges and future work

As mentioned in Section 2.6, in the present work, a single magnetometer sample per IMU is always used for initializing the IMU orientations in one common reference frame. Hence, magnetometer-free refers to not using the magnetometer model in the tracking (after initialization). A magnetometer-free initialization is therefore an open challenge and part of our future work. First work in this direction can be found in 47.

The above summarized results refer to magnetometer-free relative IMU orientation estimates, since drift-free global heading estimation without magnetometers is not in the focus of this work. Moreover, we did not focus on critical cases such as completely stationary phases or phases with motion only along the gravitational vector. In these cases, relative IMU orientations are also not observable in the proposed approach without using magnetometers ^39^. However, an intelligent usage of magnetometer information to address these cases is currently investigated.

Moreover, consistent joint position convergence depends on sufficient motion excitation and thus on the movement. In the case of insufficient motion excitation for joint position convergence, prior knowledge, e.g. a prior on the segment-length like in 20, could be included, or the joint position uncertainty could be used to guide the person to perform motions that target the uncertain degrees of freedom.

Despite the non-observability of the joint position of a perfect hinge via the proposed approach, as described in Section 3.4.1, reasonable joint position estimates were obtained for both the human and the robot dataset. At the same time, in particular for the robot with the mechanical hinges at the knees, improved results might be obtained when injecting this prior knowledge via specific hinge measurement models.

The proposed scalar joint position uncertainty measure proved to be a good indicator for joint position convergence in the experiments, in particular in the simulation study. On real data, a scaling calibration might be required as an additional step. However, the approach is not robust against outliers. In particular, imprecise or even false positive zero velocity detections (as observed in the human and robot experiments) prior to convergence decrease the uncertainty and at the same time significantly harm the joint position estimates. For locomotion scenarios, it is therefore desirable to decouple the joint position estimation from the global position drift reduction mechanism.

Also, since the covariances of the joint position estimates typically decrease with each incoming measurement, stationary phases (e.g. if the person stands still) can lead to overly certain estimates in a specific direction. This can slow down the convergence (even if it would not decrease the proposed convergence indicator).

Finally, the integration of the proposed approach with inside-out or outside-in visual pose reconstruction is part of our future work.

## Ethics and consent

The TUK-6-minute-walking dataset ^42^ study was approved by the ethical committee of the Rheinland-Pfälzische Technische Universität (RPTU), formerly known as Technische Universität Kaiserslautern (TUK) (date of approval 5.12.2017), and meets the criteria of the declaration of Helsinki. After receiving all relevant study information, the participants signed a written informed consent to the study including a permission to publish the data.

## Data Availability

Zenodo: Underlying data for ’JointTracker: Real-time inertial kinematic chain tracking with joint position estimation’, https://doi.org/10.5281/zenodo.10253111 ^42^ (TUK-6-minute-walking dataset) This project contains the following underlying data: Description.pdf BVH 6 min walking test.zip HDF5 6 min walking test.zip Zenodo: Underlying data for ’JointTracker: Real-time inertial kinematic chain tracking with joint position estimation’, https://doi.org/10.5281/zenodo.10253284 ^44^ (IMU and marker-based optical motion capture from a humanoid robot) This project contains the following underlying data: Description.pdf Robot circle run 0003.h5 Robot circle run 0004.h5 Robot turning 0003.h5 Data are available under the terms of the Creative Commons Attribution 4.0 International license (CC-BY 4.0)

## Software availability

Source code for data visualization and inspection available from: https://github.com/BTaetz/Visualizer-SkeletalMotionHDF5

License: MIT
