# Supplementary material for: JointTracker: Real-time inertial kinematic chain tracking with joint position estimation
Source: Open Res Eur. 2025 Jan 22;4:33. Originally published 2024 Feb 19. [Version 2] doi: 10.12688/openreseurope.16939.2 (PMC11216284; doi:10.12688/openreseurope.16939.2)
Supplement: Peer review history

# Reviewer report: Reviewer response for version 2

**DOI:** 10.21956/openreseurope.20759.r50175

**Published:** 2025-03-27

**Authors:** Manon Kok

**Affiliations:** 1 Delft University, Delft, The Netherlands

Thank you for the detailed rebuttal and congrats on a very nice paper!

Is the rationale for developing the new method (or application) clearly explained?

Yes

Is the description of the method technically sound?

Yes

Are the conclusions about the method and its performance adequately supported by the findings presented in the article?

Partly

If any results are presented, are all the source data underlying the results available to ensure full reproducibility?

Yes

Are sufficient details provided to allow replication of the method development and its use by others?

Yes

Reviewer Expertise:

Sensor fusion

I confirm that I have read this submission and believe that I have an appropriate level of expertise to confirm that it is of an acceptable scientific standard.

# Reviewer report: Reviewer response for version 2

**DOI:** 10.21956/openreseurope.20759.r50173

**Published:** 2025-02-04

**Authors:** Simon Bachhuber

**Affiliations:** 1 FAU Erlangen-Nuernberg, Nürnberg, Germany

Thank you for the point-by-point response and the revision of your article.

 While I believe the document would benefit from improvements in figure quality and equation typesetting, the contributions and results remain comprehensible and merit indexing. However, not open-sourcing the code seems like a missed opportunity, and the suggestion to self-implement the method is not satisfactory.

Is the rationale for developing the new method (or application) clearly explained?

Yes

Is the description of the method technically sound?

Yes

Are the conclusions about the method and its performance adequately supported by the findings presented in the article?

Partly

If any results are presented, are all the source data underlying the results available to ensure full reproducibility?

Partly

Are sufficient details provided to allow replication of the method development and its use by others?

Partly

Reviewer Expertise:

Applied Machine Learning in the field of inertial motion capture

I confirm that I have read this submission and believe that I have an appropriate level of expertise to confirm that it is of an acceptable scientific standard.

# Reviewer report: Reviewer response for version 1

**DOI:** 10.21956/openreseurope.18306.r41351

**Published:** 2024-06-28

**Authors:** Lauro Armando Contreras Rodríguez

**Affiliations:** 1 Universidad Autonoma de Sinaloa, Culiacán, Sinaloa, Mexico

Overall, the article adequately presents the development of the evaluation of an algorithm for real-time monitoring of the position of joints using inertial sensors.

 Taking into consideration the problem posed in the introduction section, the methodological process describes step-by-step the algorithm used to capture the information through the optimization of the derivation of a recursive Bayesian estimator for each IMU sensor to subsequently adapt it to a kinematical model.

 The results shown provide a clear explanation of what was obtained for the assessment scenarios, as well as the conclusions show the virtues and drawbacks of the proposal.

 However, there are some points that must be addressed:

- Section 3.1 (Evaluation metrics) provides information that describes how the proposal will be assessed, this information belongs to the methodology section.
- Figure 12, Table 4, and Table 5 correspond to the section 3.4, not the Results section.
- Section 3.3.1 (Experimental setup) requires further information related to the test persons such as: age, height, and weight. The word “young” used to describe the population of this study could lead to interpretation of the use of children, adolescents, and/or young adults. Height and weight help to estimate other movement conditions that are affected by these parameters (the angular velocity is different according to the body segment’s size and soft tissues could lead to misalignments of the IMU sensors that modify the interpretation of the body movement).
- A direct comparison between the measurements of the humanoid robot and the test persons could provide information related to the sensor's agreement for its use in both systems. Bland-Altman analysis could be used to quantify this agreement.

Is the rationale for developing the new method (or application) clearly explained?

Yes

Is the description of the method technically sound?

Yes

Are the conclusions about the method and its performance adequately supported by the findings presented in the article?

Yes

If any results are presented, are all the source data underlying the results available to ensure full reproducibility?

Yes

Are sufficient details provided to allow replication of the method development and its use by others?

Yes

Reviewer Expertise:

Biomechanical movement; IMU sensor; Sport science.

I confirm that I have read this submission and believe that I have an appropriate level of expertise to confirm that it is of an acceptable scientific standard.

# Response

**Published:** 2024-12-19

**Authors:** Bertram Taetz

**Affiliations:** Deutsches Forschungszentrum für Künstliche Intelligenz (DFKI), Germany

**We thank the reviewer for reading the article in depth and for providing detailed and helpful comments! Below, we address the review point by point, with our answers being underlined and bold. Note, the figure and reference numbers in the answers refer to version 1 (first submission).** Section 3.1 (Evaluation metrics) provides information that describes how the proposal will be assessed, this information belongs to the methodology section. **We agree that this is one option. However, in our article, the developed algorithm and its mathematical basis are considered the method and are therefore described in the methods section. The evaluation metrics are not a part of the method (algorithm) and are therefore described in the evaluation section, together with the experimental setups and results. To make this clearer, we changed the section title from “Results” to “Evaluation and results”. We believe that this is a comprehensible distribution of information over the sections and would like to keep it that way.** Figure 12, Table 4, and Table 5 correspond to the section 3.4, not the Results section. **We agree that the mentioned figure and tables belong to Section 3.4, which is a subsection of Section 3 (“Results”, now “Evaluation and Results”). Note that the figure and tables are also referenced correctly in Section 3.4.**

**Note, based on a comment from another reviewer, we removed Figure 5 and Figure 7, so that the mentioned parts should appear closer to the references in the text in the revised article.** Section 3.3.1 (Experimental setup) requires further information related to the test persons such as: age, height, and weight. The word “young” used to describe the population of this study could lead to interpretation of the use of children, adolescents, and/or young adults. Height and weight help to estimate other movement conditions that are affected by these parameters (the angular velocity is different according to the body segment’s size and soft tissues could lead to misalignments of the IMU sensors that modify the interpretation of the body movement). **We added the missing information to the article: The dataset contains synchronized lower body IMU and optical reference data from 25 young, healthy test persons (15 females, age 24.60 ± 2.70 years; 69.44 ± 12.17 kg and 1.75 ± 0.08 m in height) and two sessions (6.75 ± 2.26 days between test and retest).** A direct comparison between the measurements of the humanoid robot and the test persons could provide information related to the sensor's agreement for its use in both systems. Bland-Altman analysis could be used to quantify this agreement. **A direct comparison is not possible, due to different IMU mountings and movements between humans and robot (note, we are estimating IMU and joint trajectories). Instead, we evaluated the tracking results with the respective reference data using the same metrics, and we reported these evaluation results, e.g. in Tables 3,4 for the humans and in Tables 5,6 for the robot. The results are then summarized and discussed in Section 4.1.**

# Reviewer report: Reviewer response for version 1

**DOI:** 10.21956/openreseurope.18306.r41352

**Published:** 2024-06-18

**Authors:** Simon Bachhuber

**Affiliations:** 1 FAU Erlangen-Nuernberg, Nürnberg, Germany

This paper proposes a method for calibration-free motion capture of kinematic chains using body-attached IMUs.

 Overall, the paper is well written and the motivation clear. However, particularly concerns regarding novelty and contributions should be clarified and alternative, baseline methods should be added to the experimental validation. Specifically, addressing the following issues will help to increase the overall quality of the manuscript:

 # Content

 A) The novelty of the work is not distinctly outlined. The authors acknowledge the existence of various I2S calibration approaches but do not explicitly compare their method to these predecessors to highlight the specific advancements made. Furthermore, while there are calibration-free IHMC methods available, the authors have not explained why these were not considered suitable for the task at hand. Consequently, it remains uncertain whether this work addresses an unresolved issue or if it offers a solution that outperforms existing methods, which would necessitate a comparative analysis with alternative approaches.

 As an example, the authors should add sentences that compare the proposed approach, e.g., "Previous approaches to joint position estimation typically work offline, often also independently per joint, and require high-frequency inertial data; e.g., 32,37,38, though drift-free, typically require a higher sampling rate for obtaining accurate results as compared to approaches working on integrated states." -> [...] In contrast to these methods, our approach does not ...

 Also directly referring to the cited sentence, it seems that the authors are motivating the contribution by: 1) online-capable, 2) not independently per joint, 3) not requiring high-frequency inertial data. But then they do not properly address these points, regarding

 - 1) The ability of the proposed method to be applicable online (and in real-time?) is not further discussed in the manuscript.

 - 2) The advantage of the proposed method compared to previous work that uses a independently per joint approach is not shown because the authors then later do not compare the proposed method to alternative, baseline methods

 - 3) This work seems to be using 60Hz+ IMUs which, whilst not being very high-frequency, certainly are also not low-frequency IMUs (like 25Hz).

 B) From the problem statement, it is unclear whether or not the IMU-to-segment orientation must be known or if sensors can be attached arbitrarily.

 C) The quality and potential of figures is not yet fully utilized. Specifically, the large amount of figures make the results difficult to comprehend. The figures seem to be presented in an exhaustive manner rather than as an highlighting tool. For example, Figure 7 seems highly space inefficient. The compressed information value of the figure is too low to justify its inclusion. A more focused presentation with well chosen figures to highlight exemplary trials and rely on tables for point estimates may be advantageous, or instead of exemplary trials, the authors could plot the average error and average uncertainty over time. 

 D) It is unclear why the joint position estimates for human trials can not be validated. The authors could test the joint position estimates by placing an additional IMU to a segment with a fixed and known offset. This way the joint position estimate must be different by exactly this known offset value for the two IMUs.

 E) There is a lack of alternative, baseline methods. The authors should compare themselves to prior work. For example, for comparing the joint estimation performance, the authors could use [Seel, et al., 2012 (Ref 1)], for comparing orientation estimation performance a simple magnetometer-reliant, joint-independent baseline can be straightforwardly computed for all scenarios. Moreover, by comparing yourself to the independent baselines (in a sense that they are applied for each joint independently) you can then then discuss the difference and advantage to the proposed kinematic-model-based approach.

 F) The code for JointTracker should be made openly available.

 # Formatting, Typesetting

 A) The formatting of figures is suboptimal.

 - The figure titles use a rather unattractive underscore notation even though the authors have defined mathematical symbols for these quantities. 

 - The x-axis should be given in units of second and labeled time for a more comprehensible presentation. The sampling rate can be included in the caption.

 - The figures should be embedded into the documents as vector graphics.

 - The font sizes, tick sizes, tick labels, titles, labels are all too small and inconsistent with the manuscript font size and font.

 # Minor

 A) The authors motivate using *human* motion capture but this choice seems confusing. The proposed method is applicable to any kinematic chain, you even shows this applicability to nonhuman systems during experimental validation. Thus, why the restriction to human motion capture in the motivation?

 B) The acronym IMU seems incorrectly introduced.

 C) Some sentences are overly lengthy and hard to understand, e.g., "Previous approaches to joint position estimation typically work offline, often also independently per joint, and require high-frequency inertial data; e.g., 32,37,38, though drift-free, typically require a higher sampling rate for obtaining accurate results as compared to approaches working on integrated states." Tools like Grammarly can detect such sentences and rewrite them accordingly.

 D) Some hyperrefs are not working, e.g., "In Section 3, we also evaluate ...".

 E) The choice of reporting length scales in meters in this context seems suboptimal. A more natural choice might be centimeters, consider for example Figure 9.

Is the rationale for developing the new method (or application) clearly explained?

Yes

Is the description of the method technically sound?

Yes

Are the conclusions about the method and its performance adequately supported by the findings presented in the article?

Partly

If any results are presented, are all the source data underlying the results available to ensure full reproducibility?

Partly

Are sufficient details provided to allow replication of the method development and its use by others?

Partly

Reviewer Expertise:

Applied Machine Learning in the field of inertial motion capture

I confirm that I have read this submission and believe that I have an appropriate level of expertise to confirm that it is of an acceptable scientific standard, however I have significant reservations, as outlined above.

## References

[1] : Joint axis and position estimation from inertial measurement data by exploiting kinematic constraints.2012; 10.1109/CCA.2012.6402423 45-49 10.1109/CCA.2012.6402423

# Response

**Published:** 2024-12-19

**Authors:** Bertram Taetz

**Affiliations:** Deutsches Forschungszentrum für Künstliche Intelligenz (DFKI), Germany

**We thank the reviewer for reading the article in depth and for providing detailed and helpful comments! Below, we address the review point by point, with our answers being underlined and bold. Note, the figure and reference numbers in the answers refer to version 1 (first submission). We hope that with our revisions and explanations, the reviewer can approve the article.**

 # Content

 A) The novelty of the work is not distinctly outlined. The authors acknowledge the existence of various I2S calibration approaches but do not explicitly compare their method to these predecessors to highlight the specific advancements made. Furthermore, while there are calibration-free IHMC methods available, the authors have not explained why these were not considered suitable for the task at hand. Consequently, it remains uncertain whether this work addresses an unresolved issue or if it offers a solution that outperforms existing methods, which would necessitate a comparative analysis with alternative approaches.

 As an example, the authors should add sentences that compare the proposed approach, e.g., "Previous approaches to joint position estimation typically work offline, often also independently per joint, and require high-frequency inertial data; e.g., 32,37,38, though drift-free, typically require a higher sampling rate for obtaining accurate results as compared to approaches working on integrated states." -> [...] In contrast to these methods, our approach does not ...

 Also directly referring to the cited sentence, it seems that the authors are motivating the contribution by: 1) online-capable, 2) not independently per joint, 3) not requiring high-frequency inertial data. But then they do not properly address these points, regarding

 - 1) The ability of the proposed method to be applicable online (and in real-time?) is not further discussed in the manuscript.

 - 2) The advantage of the proposed method compared to previous work that uses a independently per joint approach is not shown because the authors then later do not compare the proposed method to alternative, baseline methods

 - 3) This work seems to be using 60Hz+ IMUs which, whilst not being very high-frequency, certainly are also not low-frequency IMUs (like 25Hz).

**We agree that our contribution was not formulated clearly enough, and that the description of previous work on joint position estimation was too condensed. Our work addresses an unresolved issue (rather than offering a solution that outperforms existing methods), since it is, to the best of our knowledge, the first real-time capable method for simultaneous joint (or fixed) position and IMU kinematics estimation for general kinematic chains (as mentioned in Section 4.1). We modified the formulation of the contributions at the end of Section 1 accordingly. Moreover, in the following, we answer to the numbered list above:**

1.
**Real-time capable here means that the estimation is recursive and uses only the data of the current time step, rather than being a batch approach (offline) or using a moving horizon with a window size > 1. The mathematical derivation in Section 2.3 addresses this from a theoretical perspective. This is now also clearly stated in Section 4.1. Moreover, we added exemplary processing times for the three-IMU and seven-IMU setup on the hardware running the experiments.**

2.
**As mentioned above, it is not our aim to outperform independently per joint methods (concerning joint position estimation), or independently per IMU orientation estimation methods, but to enable online simultaneous estimation of the states of a moving skeleton (or kinematic chain), plug and play, and with only minimal magnetometer usage, which was a previously unresolved issue. By using our approach, we can obtain consistent relative positional (and rotational) information between all the joints, so that the overall pose of the kinematic chain can be visualized in 3D (by connecting the joint centers, similar to how it is done based on markerless camera-based tracking). The latter is now also explained at the end of Section 1.**

3.
**We agree that “require a higher sampling frequency” was not sufficiently analyzed in the article, and we therefore removed this formulation. The more important aspect is that integrated states (velocities and positions), as used in our proposed algorithm (Section 2.5.2), provide drift correction on IMU position and velocity level, which is required to obtain an overall consistent kinematic tracking, and that they provide smoother signals compared to accelerations (as used for joint position estimation in the mentioned previous work).**

**We modified the paragraph about previous work on joint position estimation in Section 1 accordingly. We also added a comparison to the acceleration-level joint connection model (in our algorithmic framework) to the experimental evaluation.**

 B) From the problem statement, it is unclear whether or not the IMU-to-segment orientation must be known or if sensors can be attached arbitrarily. 

**The IMU-to-segment orientation is not required. This is now more clearly stated at the end of Section 2.1. The sensors can be attached arbitrarily.**

 C) The quality and potential of figures is not yet fully utilized. Specifically, the large amount of figures make the results difficult to comprehend. The figures seem to be presented in an exhaustive manner rather than as an highlighting tool. For example, Figure 7 seems highly space inefficient. The compressed information value of the figure is too low to justify its inclusion. A more focused presentation with well chosen figures to highlight exemplary trials and rely on tables for point estimates may be advantageous, or instead of exemplary trials, the authors could plot the average error and average uncertainty over time. 

**We agree that there is a rather large amount of figures in the article, even though we had reduced them already before the first submission. However, we believe that most of the selected figures are needed to demonstrate the convergence behaviors, uncertainties and temporal progressions of estimates. For Figure 7, we removed this and included the averaged results in the main text. We also removed Figure 5 (Three-link chain: Angle errors and init uncertainties of estimated relative IMU orientations), since it is very similar to Figure 6 (same with global IMU orientations).**

 D) It is unclear why the joint position estimates for human trials can not be validated. The authors could test the joint position estimates by placing an additional IMU to a segment with a fixed and known offset. This way the joint position estimate must be different by exactly this known offset value for the two IMUs. 

**We agree that the method described by the reviewer could be used for validation. However, in experimental setup 2, we worked with an existing dataset containing test and retest measurements from 25 test persons, and we believe that it is an interesting evaluation to analyze reliability based on this, in addition to the other two validation experiments based on simulated data and the robot. With this, we cover both, validity and reliability. Also note that the article doesn’t state that joint position estimates for human trials cannot be validated in general, just that we do not have exact ground truth data for the available dataset and focus our analysis on reliability. Please excuse if we miss something.**

 E) There is a lack of alternative, baseline methods. The authors should compare themselves to prior work. For example, for comparing the joint estimation performance, the authors could use [Seel, et al., 2012 (Ref 1)], for comparing orientation estimation performance a simple magnetometer-reliant, joint-independent baseline can be straightforwardly computed for all scenarios. Moreover, by comparing yourself to the independent baselines (in a sense that they are applied for each joint independently) you can then then discuss the difference and advantage to the proposed kinematic-model-based approach. 

**As explained above (A), it is not our aim to outperform independently per joint or offline methods, but to enable a new functionality (online, calibration-free, kinematic chain tracking (simultaneous IMU kinematics and joint positions)), and to demonstrate that the proposed algorithm works in principle (using simulated data in experiment 1) and under different realistic and partly suboptimal conditions (using data from humans and a robot in experiments 2 and 3). However, to address this comment, as also mentioned above, we added a comparison of the proposed approach (using joint connection measurement models on positional and velocity level) with a variant using an acceleration level joint connection measurement model as proposed in [38], for the simulated data (three-link chain). This demonstrates that our algorithmic framework operating on a complete kinematic chain requires working on integrated states (position and velocity), since significantly better results are obtained.**

 F) The code for JointTracker should be made openly available. 

**The implementation of JointTracker is integrated (in a distributed way) into a bigger C++ software library. Unfortunately, there are no resources available to isolate the code and make it openly available. However, the article includes a detailed mathematical description of the algorithm, and the datasets have been made available. To nevertheless address this comment, we could offer an executable of JointTracker on individual request of other researchers.**

 # Formatting, Typesetting

 A) The formatting of figures is suboptimal.

 - The figure titles use a rather unattractive underscore notation even though the authors have defined mathematical symbols for these quantities. 

 - The x-axis should be given in units of second and labeled time for a more comprehensible presentation. The sampling rate can be included in the caption.

 - The figures should be embedded into the documents as vector graphics.

 - The font sizes, tick sizes, tick labels, titles, labels are all too small and inconsistent with the manuscript font size and font.

**We agree that the formatting of the figures could be optimized. However, we believe that the figures and figure texts (titles, ticks, labels) are in their current form reasonably well readable, comprehensible and consistent with respect to each other (none of the other reviewers commented on this). We therefore hope for the reviewer’s understanding of our decision to keep the figures as they are and invest our efforts into addressing the other comments.**

 # Minor

 A) The authors motivate using *human* motion capture but this choice seems confusing. The proposed method is applicable to any kinematic chain, you even shows this applicability to nonhuman systems during experimental validation. Thus, why the restriction to human motion capture in the motivation?

**The emphasis on human motion capture as motivation comes mainly from the fact that most (previous) motion capture research is in the field of human motion capture. However, it is a valid comment, and we reduced the emphasis on “human” in the abstract as well as in the introduction (beginning of Section 1). We also added the following sentence to Section 1 “The model is applicable to any kinematic chain and not restricted to or specialized to a human skeleton.” (which is also an advantage of the proposed method over [34,35])**

 B) The acronym IMU seems incorrectly introduced. 

**We introduced it now as “inertial measurement unit” in the second paragraph of Section 1.**

 C) Some sentences are overly lengthy and hard to understand, e.g., "Previous approaches to joint position estimation typically work offline, often also independently per joint, and require high-frequency inertial data; e.g., 32,37,38, though drift-free, typically require a higher sampling rate for obtaining accurate results as compared to approaches working on integrated states." Tools like Grammarly can detect such sentences and rewrite them accordingly. 

**We modified this sentence.**

 D) Some hyperrefs are not working, e.g., "In Section 3, we also evaluate ...". 

**This specific hyperlink seems to work in the PDF and DOC we downloaded. However, we found other incorrect hyperlinks, which we corrected (Section 2.2., references to equations (49), (50)).**

 E) The choice of reporting length scales in meters in this context seems suboptimal. A more natural choice might be centimeters, consider for example Figure 9. 

**We agree that centimeters could have been a better choice and will consider this for future submissions. However, since all positional results are consistently reported in meters (figures and tables), we would like to keep it in this article.**

# Reviewer report: Reviewer response for version 1

**DOI:** 10.21956/openreseurope.18306.r39589

**Published:** 2024-05-09

**Authors:** Manon Kok

**Affiliations:** 1 Delft University, Delft, The Netherlands

The paper presents a method for recursive inertial human motion estimation in which the joint position is estimated simultaneously with the pose, thereby resulting in a calibration-free method. The paper presents an interesting approach and recursive, calibration-free motion estimation is an important topic. According to me the contribution is definitely worth publishing but I think the presentation needs improvement before doing so. I specifically think the contribution needs to be presented more clearly throughout the whole paper. More specifically: 

 1) The contribution is not clear from the abstract and introduction. More specifically: 

 a) Both the abstract and the introduction first seem to suggest the paper is about a camera-based approach, while it is actually about an inertial sensor-based approach. To aid the clarity to a reader, I would make this clear right from the start of the paper.

 b) The last sentence of the abstract suggests that the presented approach can be used in visual-inertial human motion capture. There are no results included related to this though, so I would suggest to only mention this as a direction of future work.

 c) In section 1, the sentences “This work contributes to … e.g. 20” are not very clear. For instance, it is not clear what “contributes to” exactly means (since it doesn’t specify that you present an algorithm), it is not clear what type of kinematic model you mean and what you refer to with visual 3D joint tracking (optitrack since that’s what you evaluate against?).

 d) You estimate joint positions. It is not uncommon that this wording is used for joint angles but you actually mean the position (in meters), right? This may be worth specifying clearly. 

 2) Also in Section 2 the contribution is not completely clear. More specifically: 

 a) In the problem statement you introduce your estimation problem but while your contribution is a recursive estimator your problem statement presents a batch approach. 

 b) Section 2.3 until Equation (32) presents the time update of your filter. This is a very long derivation which as far as I can see ends up with a standard Kalman filter update? If this is the case, I would suggest to just present your approach much more concisely just as an iterated EKF. I think this will help the reader and hence increase the impact of the work and the theoretical presentation in terms of an optimization problem and the limits can be included in an appendix. 

 c) You mention below Equation (11) that the states X_t^2 do not have an associated measurement model. This is not clear at that time in the text since it’s not yet known what measurement models you use. 

 d) In Section 2.5 it’s not clear that you test your algorithm with and without some measurement models (e.g. the magnetometer) but that some of them you include in all tests. 

 e) The fixed positions that you describe in Section 2.5.3 are not clear, especially also not since I didn’t see them mentioned anywhere in Section 3. 

 e) Only at the end of section 2.1 you mention “Joint Tracker” but this has not been introduced anywhere. In fact, throughout the paper you refer to your method at Joint Tracker, an iterated EKF and an optimization-based method. I would suggest to stick to one naming. 

 f) You mention I2S calibration multiple times, e.g. at the end of section 2.1 and in Equation (59). It is not clear how this relates to the estimates from your approach as you don’t estimate the relative orientation of the sensor with respect to the segments?  

 g) The title of section 2.6 is observability and identifiability but throughout the text you only mention observability. In Section 3.2 you use the wording identifiability though. 

 3) The relation between your main contribution and the results is also not completely clear: 

 a) It is not clear what you want to show exactly with the experiments. For instance, you test a lot how global / relative orientation estimates depend on including the magnetometer or not. But in the rest of the paper it is not clear that this is an important part of your study. 

 b) To me the legend entries “init uncertainty” and “max model uncertainty” are not clear. With init I expect that this is related to the initialization which I don’t think is the case. And why don’t you refer to the max model uncertainty as your uncertainty measure? 

 c) Table 2 shows very large rotation errors but I don’t think this is discussed in the text? 

 d) 10 Monte Carlo simulations seems very little. 

 e) How does your method handle biases in the inertial sensor signals? 

 f) Why did you correctly initialize the positions and poses in Section 3.2.4?

 4) Some comments about the conclusions: 

 a) You conclude that your method can be run real-time, but this is not evaluated in Section 3 (no computational times are included). 

 b) You also draw a conclusion about convergence speed, but this is highly dependent on the movement, right? 

 c) You mention that you obtain reasonable estimates of unobservable variables. Why is this the case and why does constraining unobservable variables lead to improved estimates? 

 5) Also a more general comment: You make an interesting design choice in Section 2 and I think it’s worth commenting on the reason for that. You choose to not include process noise on the position and velocity states but then you use a “loose” joint constraint for both the position and the velocity in Section 2.5.2. Why do you not take the limit of those noises to zero as well? And why do you need a constraint on both if both are completely linked through the zero process noise in the time update? 

 6) Some additional comments: 

 a) The formatting of the paper is not intuitive to me, though of course it could be part of the template: Whenever there are numbers in the paper, these are citations. I would expect some brackets around these. Furthermore, e.g. on page 4 (2.1) refers to a section while I would expect this to be a reference to an equation. 

 b) Some parts of equations / matrices, e.g. in Equations (2) and (3) are highlighted. It is not clear why. 

 c) Shouldn’t it be argmax instead of max in Equations (5) and (7)? 

 d) Shouldn’t the epsilon three lines below (18) be a different symbol than the epsilon of which you just took the limit to zero? 

 e) There’s a dot missing on the I in Equation (8) in the definition of X^2. 

 f) The notation of the exp and the quaternion multiplication in (42) have not been introduced. 

 g) Was E_{l,t} in (58) defined? 

 h) In Figure 7 your legend suggests that there are yellow dots in the figure that are barely visible. The red dots, however, are not mentioned in the legend.

Is the rationale for developing the new method (or application) clearly explained?

Yes

Is the description of the method technically sound?

Yes

Are the conclusions about the method and its performance adequately supported by the findings presented in the article?

Partly

If any results are presented, are all the source data underlying the results available to ensure full reproducibility?

Yes

Are sufficient details provided to allow replication of the method development and its use by others?

Yes

Reviewer Expertise:

Sensor fusion

I confirm that I have read this submission and believe that I have an appropriate level of expertise to confirm that it is of an acceptable scientific standard, however I have significant reservations, as outlined above.

# Response

**Published:** 2024-12-19

**Authors:** Bertram Taetz

**Affiliations:** Deutsches Forschungszentrum für Künstliche Intelligenz (DFKI), Germany

We thank the reviewer for reading the article in depth and for providing detailed and helpful comments! Below, we address the review point by point, with our answers being underlined. Note, the figure and reference numbers in the answers refer to version 1 (first submission). We hope that with our revisions and explanations, the reviewer can approve the article.

 1) The contribution is not clear from the abstract and introduction. More specifically: 

 a) Both the abstract and the introduction first seem to suggest the paper is about a camera-based approach, while it is actually about an inertial sensor-based approach. To aid the clarity to a reader, I would make this clear right from the start of the paper. 

**Response:** True, this was driven by our current research to fuse the JointTracker results with markerless visual approaches, which also typically detect joint positions in the images and lift them to 3D. However, this is work in progress and shouldn’t be over-emphasized in this article. Hence, we removed the state-of-the-art of visual body tracking in the introduction and reduced it in the abstract.

 b) The last sentence of the abstract suggests that the presented approach can be used in visual-inertial human motion capture. There are no results included related to this though, so I would suggest to only mention this as a direction of future work.

**Response:** We removed this part from the abstract.

 c) In section 1, the sentences “This work contributes to … e.g. 20” are not very clear. For instance, it is not clear what “contributes to” exactly means (since it doesn’t specify that you present an algorithm), it is not clear what type of kinematic model you mean and what you refer to with visual 3D joint tracking (optitrack since that’s what you evaluate against?).

**Response:** We modified this paragraph to be clearer. We specified that we contribute an algorithm and its derivation. The kinematic model is shown in Figure 1 (left). It is represented by global IMU poses with local (IMU-centered) joint positions. We further explained the markerless visual 3D joint tracking approaches we are referring to.

 d) You estimate joint positions. It is not uncommon that this wording is used for joint angles but you actually mean the position (in meters), right? This may be worth specifying clearly. 

**Response:** Right, we specified this clearly in Section 1.

 2) Also in Section 2 the contribution is not completely clear. More specifically: 

 a) In the problem statement you introduce your estimation problem but while your contribution is a recursive estimator your problem statement presents a batch approach. 

**Response:** The batch approach is first presented, since the recursive estimator is derived from this. This is now more clearly explained at the beginning of Section 2. All mentioned previous work on joint position estimation uses a batch approach.

 b) Section 2.3 until Equation (32) presents the time update of your filter. This is a very long derivation which as far as I can see ends up with a standard Kalman filter update? If this is the case, I would suggest to just present your approach much more concisely just as an iterated EKF. I think this will help the reader and hence increase the impact of the work and the theoretical presentation in terms of an optimization problem and the limits can be included in an appendix. 

**Response:** We understand this suggestion. In fact, we had included the mathematical derivation in the appendix to start with (before submission), both for the batch and the recursive estimator. Internal feedback from other researchers in the field suggested that we should include the derivation into the main body, since it represents a theoretical contribution in terms of an extension of [42]. We would therefore like to keep the derivation in the main text, and we added an explanation to the beginning of Section 2.

 c) You mention below Equation (11) that the states X_t^2 do not have an associated measurement model. This is not clear at that time in the text since it’s not yet known what measurement models you use. 

**Response:** We added a forward reference to the measurement model section.

 d) In Section 2.5 it’s not clear that you test your algorithm with and without some measurement models (e.g. the magnetometer) but that some of them you include in all tests. 

**Response:** We added a sentence at the beginning of Section 2.5, explaining that the magnetometer measurement model is optional and its effects are evaluated in the article.

 e) The fixed positions that you describe in Section 2.5.3 are not clear, especially also not since I didn’t see them mentioned anywhere in Section 3. 

**Response:** The fixed positions measurement model is used for the simulation study on the three-link chain. This is explained at the beginning of Section 3.2 “This section presents the evaluation results of the proposed approach using the fixed position measurement model for positional drift compensation (see Section 2.5.3) on simulated (noisy) IMU data”. For the other two experiments, the zero velocity detections and measurement model are used instead, since these movements involve locomotion. This is also explained at the beginning of Section 2.5 “In this work, we evaluate two optional measurement models for positional drift compensation: 1) fixed position (assuming that   one IMU moves around a fixed point in the navigation frame) is evaluated on the simulated three link chain (see Section 3.2), 2) zero velocity detections are evaluated on the lower body walking data from humans (see Section 3.3) and a humanoid robot (see Section 3.4). Note, for the purpose of positional drift compensation, position or velocity based information needs to be induced into the estimation at least for one IMU, since the joint connection models ((49, 50) below) ensure the propagation of such induced information through the kinematic chain”. Note, for the fixed position in the world-frame (P^N_fix), an arbitrary 3D coordinate can be assumed (e.g. the origin).

 e) Only at the end of section 2.1 you mention “Joint Tracker” but this has not been introduced anywhere. In fact, throughout the paper you refer to your method at Joint Tracker, an iterated EKF and an optimization-based method. I would suggest to stick to one naming. 

**Response:** We replaced “JointTracker” with “proposed algorithm”.

 f) You mention I2S calibration multiple times, e.g. at the end of section 2.1 and in Equation (59). It is not clear how this relates to the estimates from your approach as you don’t estimate the relative orientation of the sensor with respect to the segments?

**Response:** Right, for the proposed algorithm, the I2S calibrations are irrelevant (neither estimated nor assumed given). We extended the explanations at the end of Section 2.1 to clarify this. In (59), the I2S calibrations are those used for simulation to generate the test IMU data, but they are not used in the algorithm. This was also added to the text.

 g) The title of section 2.6 is observability and identifiability but throughout the text you only mention observability. In Section 3.2 you use the wording identifiability though. 

**Response:** We used the term identifiability in the context of the joint and fixed positions, since these can be considered as parameters of the system. However, since these quantities are estimated as part of the state together with the IMU kinematics in the final algorithm, observability is the correct term to use, and we replaced all mentions of identifiability with observability.

 3) The relation between your main contribution and the results is also not completely clear: 

 a) It is not clear what you want to show exactly with the experiments. For instance, you test a lot how global / relative orientation estimates depend on including the magnetometer or not. But in the rest of the paper it is not clear that this is an important part of your study. 

**Response:** As mentioned above, we revised the formulation of the contributions. In summary, these are: mathematical derivation of real-time capable state estimator, calibration-free simultaneous online IMU kinematics and joint (or fixed) position estimation for kinematic chains (leads to segment length estimation for all segments connecting two joints), convergence indicator for the motion-dependent joint position  parameters, minimal magnetometer usage (only for initialization). The above-mentioned tests with and without magnetometer measurement model activation address the last bullet point.

 b) To me the legend entries “init uncertainty” and “max model uncertainty” are not clear. With init I expect that this is related to the initialization which I don’t think is the case. And why don’t you refer to the max model uncertainty as your uncertainty measure? 

**Response:** The term “init uncertainty” was chosen, since it represents the uncertainty due to different joint initializations from the Monte Carlo sampling. We extended the explanation in Section 3.2.2. Moreover, in Section 3.2.2, we explain, that the max model uncertainty is used as uncertainty measure: “The latter is our currently proposed scalar joint position uncertainty measure to indicate the motion excitation for the respective joint.” We added the sentence: “Note, during online estimation, we have no Monte Carlo samples, and the uncertainty is based on the estimation covariance.”

 c) Table 2 shows very large rotation errors but I don’t think this is discussed in the text? 

**Response:** The numbers in Table 2 are not our evaluation results, but they quantify the IMU mounting differences between the test and retest data in the TUK-6-minute-walking dataset, as explained in Section 3.3.2. We further clarified this in the text. This shows that the IMU data was different per person between test and retest (among other things, due to different IMU mountings), while the differences in estimated segment lengths were quite small, which speaks for the reliability of the proposed algorithm.

 d) 10 Monte Carlo simulations seems very little. 

**Response:** In a related offline approach [34], which is mentioned in the introduction, evaluation was performed with only one initialization (no Monte Carlo sampling) using assumptions: “Static vectors from IMUs to neighboring joint centers and knee axes were initialized from an assumed nominal alignment.”. Compared to this, we use a zero initialization of the joint centers (without assumptions), with additional 10 offset Monte Carlo samples, as explained in Section 2.6. Together with the variety of test sequences (1 simulated+25 test persons+1 robot), we believe that this amount of sampling can be considered a reasonable trade-off between computational efforts and sampling variance.

 e) How does your method handle biases in the inertial sensor signals? 

**Response:** This information was indeed missing. We added the following sentence to the beginning of Section 3: “Sensor biases were subtracted from the real IMU data as a pre-processing step.” As mentioned in Section 2.2, the proposed algorithm can be extended for biases, but we didn’t focus on this in this article: “Note, additional parameter estimates, like sensor biases, often denoted as θ40, could be included, but are  left out for brevity of notation”.

 f) Why did you correctly initialize the positions and poses in Section 3.2.4? 

**Response:** We did this to specifically test for long-term stability after convergence. This explanation was added to the article.

 4) Some comments about the conclusions: 

 a) You conclude that your method can be run real-time, but this is not evaluated in Section 3 (no computational times are included). 

**Response:** The conclusion states “real-time capable” and “promising method for real-time”, since, by design, the proposed algorithm uses only the data of the current timestep. This explanation was added to Section 4.1. Moreover, we added exemplary processing times for the three-IMU and seven-IMU setup on the hardware running the experiments.

 b) You also draw a conclusion about convergence speed, but this is highly dependent on the movement, right? 

**Response:** This is correct. In Section 4.1, we do not draw general conclusions concerning the convergence speed, but in relation to the different experimental setups. To make the dependence on the movement even clearer, we extended the explanation in Section 4.2: “Moreover, consistent joint position convergence depends on sufficient motion excitation and thus on the movement. In the case of insufficient motion excitation for joint position convergence, prior knowledge, e.g. a prior on the segment-length like in 34, could be included, or the joint position uncertainty could be used to guide the person to perform motions that target the uncertain degrees of freedom.”

 c) You mention that you obtain reasonable estimates of unobservable variables. Why is this the case and why does constraining unobservable variables lead to improved estimates? 

**Response:** We changed “constraining” to “adding prior knowledge”, i.e.: “At the same time, in particular for the robot with the mechanical hinges at the knees, improved results might be obtained when injecting this prior knowledge via specific hinge measurement models.” We have an initial implementation of such a specific hinge measurement model, which we; however, didn’t include in this article, since 1) the article would become too long, 2) the measurement model didn’t bring significant improvements for the considered datasets (only for a perfectly simulated hinge joint) **.**

 5) Also a more general comment: You make an interesting design choice in Section 2 and I think it’s worth commenting on the reason for that. You choose to not include process noise on the position and velocity states but then you use a “loose” joint constraint for both the position and the velocity in Section 2.5.2. Why do you not take the limit of those noises to zero as well? And why do you need a constraint on both if both are completely linked through the zero process noise in the time update? 

**Response:** The measurement noises for the joint connection measurement models are required to be able to cast the estimation problem as least squares estimation (and estimate the joint positions as part of the state). Otherwise, the connection model must be formulated with additional Lagrange multipliers to obtain a constrained optimization problem. This, however, increases computational complexity and therefore processing time.  It is true that the velocity measurement model could be left out from a theoretical point of view. However, during empirical tests on the three-link chain, we observed a slower convergence and slightly lower accuracy when leaving it out. We added this explanation to Sections 2.5.2 and 3.2.2.

 6) Some additional comments: 

 a) The formatting of the paper is not intuitive to me, though of course it could be part of the template: Whenever there are numbers in the paper, these are citations. I would expect some brackets around these. 

**Response:** Brackets were included in our initial submission, but they were removed during the production process of the article.

 Furthermore, e.g. on page 4 (2.1) refers to a section while I would expect this to be a reference to an equation. 

**Response:** We added “Section” in front of these numbers.

 b) Some parts of equations / matrices, e.g. in Equations (2) and (3) are highlighted. It is not clear why. 

**Response:** The highlighting came from the Latex template used for initial submission, and we were not able to remove it. We asked, whether this could be removed in the revision.

 c) Shouldn’t it be argmax instead of max in Equations (5) and (7)? 

**Response:** Yes, these are mistakes! We corrected them.

 d) Shouldn’t the epsilon three lines below (18) be a different symbol than the epsilon of which you just took the limit to zero? 

**Response:** No, this is correct, since ϵ does not reach zero.

 e) There’s a dot missing on the I in Equation (8) in the definition of X^2. 

**Response:** Thanks, we corrected this.

 f) The notation of the exp and the quaternion multiplication in (42) have not been introduced. 

**Response:** We added an introduction of this notation in Section 2.4.

 g) Was E_{l,t} in (58) defined? 

**Response:** The l is explained below (58) and E can denote an error, such as the ones defined in (56), (57). We added an explicit explanation for this in Section 3.1.

 h) In Figure 7 your legend suggests that there are yellow dots in the figure that are barely visible. The red dots, however, are not mentioned in the legend.

**Response:** We  adapted the caption in Figure 8 (Figure 7 was removed based on a comment from another reviewer). The light orange dots appear red over the blue dots.
